# From Spontaneous Motor Activity to Coordinated Behaviour: A Developmental Model

**DOI:** 10.1371/journal.pcbi.1003653

**Published:** 2014-07-24

**Authors:** Hugo Gravato Marques, Arjun Bharadwaj, Fumiya Iida

**Affiliations:** Dept. of Mechanical and Process Engineering, ETH, Zurich, Switzerland; Indiana University, United States of America

## Abstract

In mammals, the developmental path that links the primary behaviours observed during foetal stages to the full fledged behaviours observed in adults is still beyond our understanding. Often theories of motor control try to deal with the process of incremental learning in an abstract and modular way without establishing any correspondence with the mammalian developmental stages. In this paper, we propose a computational model that links three distinct behaviours which appear at three different stages of development. In order of appearance, these behaviours are: spontaneous motor activity (SMA), reflexes, and coordinated behaviours, such as locomotion. The goal of our model is to address *in silico* four hypotheses that are currently hard to verify *in vivo*: First, the hypothesis that spinal reflex circuits can be self-organized from the sensor and motor activity induced by SMA. Second, the hypothesis that supraspinal systems can modulate reflex circuits to achieve coordinated behaviour. Third, the hypothesis that, since SMA is observed in an organism throughout its entire lifetime, it provides a mechanism suitable to maintain the reflex circuits aligned with the musculoskeletal system, and thus adapt to changes in body morphology. And fourth, the hypothesis that by changing the modulation of the reflex circuits over time, one can switch between different coordinated behaviours. Our model is tested in a simulated musculoskeletal leg actuated by six muscles arranged in a number of different ways. Hopping is used as a case study of coordinated behaviour. Our results show that reflex circuits can be self-organized from SMA, and that, once these circuits are in place, they can be modulated to achieve coordinated behaviour. In addition, our results show that our model can naturally adapt to different morphological changes and perform behavioural transitions.

## Author Summary

Mammals display a fascinating behavioural proficiency, which is a remarkable feature given the number of muscles that need to be continuously coordinated. Understanding the processes that give rise to this level of performance has been the main focus of many researchers during the last century, but until now a comprehensive model is yet to be established. From a theoretical point of view, we believe that a key element to understanding mammalian behaviour lies in processes that occur during early development. During this stage, a great deal of information is laid down in neural circuits about the structure of the musculoskeletal system. We believe that this information is essential to achieve coordinated behaviour. In this paper, we propose a model that links three behaviours that appear at different stages of mammalian development. In order of appearance these behaviours are: spontaneous motor activity, spinal reflexes, and coordinated behaviours. Our model shows that the sensor and motor activity induced by spontaneous motor activity is sufficient to self-organize spinal reflexes. These circuits enclose core knowledge about the muscle interactions occurring in the musculoskeletal system. In addition, our model shows that coordinated behaviour can then be achieved simply by modulating the reflex circuits.

## Introduction

In mammals, the developmental path that links the rudimentary behaviours observed during foetal stages to the full fledged behaviours observed in adults is still beyond our understanding [1]. We observe foetus generate spontaneous motor activity [2], [3], we observe newborns react reflexively to external stimulation, and later in life we observe adults skilfully strolling around. The period of time that goes from the first stage to the last can be longer (e.g. in altricial species) or shorter (e.g. precocial species), but all mammals undergo this general developmental path. It is noteworthy that in this paper we restrict the scope of the term *mammal* to refer only to terrestrial mammals.

It is commonly agreed that mammalian development is intrinsically incremental. Intuitively, this notion fits well with natural observations; simpler behaviours, like reflexes, tend to appear first, and only after these are in place, can one observe more elaborated and purposeful ones, like locomotion. Whether the presence of the former is required for the execution of the latter has been historically disputed [4]–[6], but nowadays the contribution of reflexes to different coordinated behaviours is widely accepted [7]–[10].

When one looks at the circuitry of the most basic reflexes, one can appreciate their close relation to the underlying morphology of the musculoskeletal system. For example, the stretch reflex [11] p.439–40, which deals with muscle-length information, entails excitatory connections with synergist *α*-motoneurons and inhibitory connections with antagonist *α*-motoneurons (see below). This symmetrical relation in the reflex circuitry mirrors a mechanical (and geometrical) relation in the musculoskeletal system: When one muscle stretches, its synergists are elongated while its antagonists are shortened. This is valid for muscle interactions at the legs as well as at the arms and torso, which could justify the invariant pattern of connectivity observed in different parts of the spinal cord. The symbiosis between reflex circuits and body morphology can as well be argued for other reflexes, like the spinal withdrawal reflex [12], or the non-spinal vestibular, auditory, and pupillary reflexes.

The proximity between reflexes and body morphology make the former ideal mechanisms to coordinate muscles at a local level. Sherrington, one of the first to recognize this relation, hypothesised that reflexes were more than stereotyped reactive responses; they were best seen as modular mechanisms that can be combined to achieve general motor coordination [5], [13]. This hypothesis was further developed into what became known as the threshold control theory or TCT, first known as the equilibrium point (EP) hypothesis [14], [15]. According to this theory, behaviour is the outcome of shifts in the equilibrium state between the organism and its environment (see also Thelen's dynamic systems theory [16]). Equilibrium shifts can be caused voluntarily by the organism, or involuntarily by the environment [15]. At the voluntary level, the nervous system can modify the current EP by shifting the reference length value of different muscles; this induces activity in the muscle spindles and, through tunable reflex circuits, produces forces that bring the organism to a new EP.

In this context, a particularly recent groundbreaking work has been that of Geyer and Herr [17]. They have shown that the motor coordination necessary to achieve stable walking in a musculoskeletal system can be brought about by tuning appropriately a number of reflexive feedback loops. However, to achieve this they have to manually establish and tune a large number of reflex circuits, which makes search for an appropriate set of parameters very difficult. A similar approach has been applied to a real-world robot, the RunBot, which can display smooth walking patterns through the coordination of reflex networks [18].

An alternative approach to the modularity of the motor system has been offered by the framework of muscle synergies [19]–[23]. This framework tries to resolve the problem of controlling a large number of degrees of freedom [24] by combining a small number of synergies (or modules), which already incorporate basic muscle activation profiles. In general, “a module is a functional unit in the spinal cord that generates a specific motor output by imposing a specific pattern of muscle activations” [25]. Modules are combined by supraspinal systems to produce the muscle activations necessary to achieve a desired task. Similarly to reflexes, synergies also seem to strongly reflect the bodies mechanical constraints [21]. At the moment, this form of modularity is formulated in a rather abstract and mathematical sense, which neglects for the most part the neural circuits as well as the developmental processes necessary to implement them (see for example [20], [22]).

**Figure 1:**
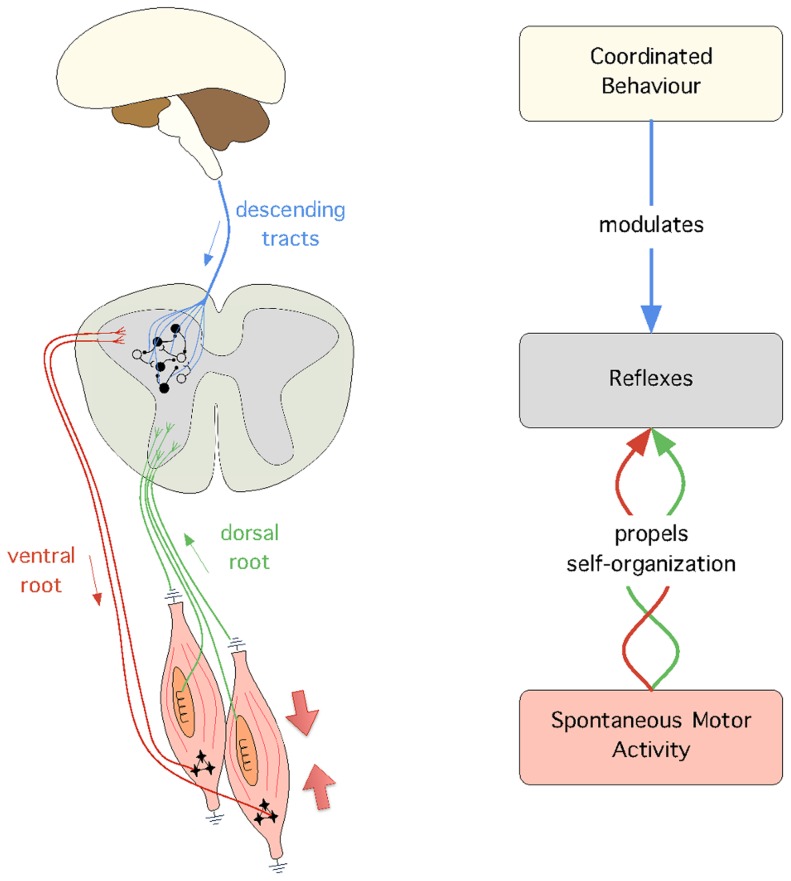
The conceptual model used in this paper. On the left are the biological mechanisms that support the model: (1) SMA is illustrated by the muscle contraction (large arrows), (2) the spinal reflex circuits, which mediate afferent (green) and efferent (red) connections, and (3) the descending signals from supraspinal circuits (blue), which modulate the activity of reflex circuits. Unfilled and filled circles illustrate the presence of excitatory and inhibitory reflex circuits, respectively. On the right, is the general model with the abstracted biological mechanisms as well as the processes that link them together, i.e. self-organization and modulation.

From a theoretical perspective, we adopt the view that development is a key aspect to understand how the nervous system achieves coordinated behaviour [26]–[28]. Following this view, this paper proposes a computational model that links three behaviours which appear at different stages of development; in order of appearance these behaviours are: spontaneous motor activity (SMA), reflexes, and coordinated behaviour (see Figure 1). The model proposed identifies the mechanisms according to which (1) SMA propels the self-organization of adaptive reflex circuits, and (2) reflex circuits are manipulated to achieve coordinated behaviour. The main motivation to build our model is to validate *in silico* four hypotheses that are currently very difficult to verify *in vivo*. First, we hypothesise that SMA induces sensory and motor responses which are sufficient to self-organize reflex circuits. This has been shown *in vivo* in the case of the spinal withdrawal reflex [29], but has not yet been established for other reflexes. Second, we hypothesise that, once meaningful reflex circuitry is in place, it can be modulated to achieve coordinated behaviour. Third, we hypothesise that, since SMA is observed after birth, throughout the entire lifetime of an individual, it provides a mechanism to continuously adapt the reflex circuits to potential morphological changes (e.g. due to injury or growth). And fourth, we hypothesise that we can achieve behavioural transitions (i.e. switch between different behaviours) by changing the modulation of the reflex gains over time.

All the experiments have been carried out in a simulated musculoskeletal leg model. As a case study for coordinated behaviour we use vertical hopping. Hopping is a particularly convenient behaviour in the context of this paper. First, it requires the activity of several muscles to be coordinated over time. Second, it requires only a single leg, which bypasses the need to deal with the problem of inter-limb coordination at this stage (see Discussion). Third, the behaviour is not limited to point-to-point movements, but it requires highly dynamical interactions between the leg and the environment (in particular the ground). And forth, it includes motion patterns that can be periodic as well as aperiodic (e.g. starting hopping from the ground). At the end of the paper, we also include an experiment in which we show how our model scales to achieve point-to-point behaviours.

The reminder of this paper is organized as follows. The second section provides the different components of our developmental model. The third section describes our experiments and results. The fourth section discusses the results obtained.

## Models

To verify our hypotheses we built a developmental model that is carried out in two subsequent stages. In the first stage we self-organize the reflex circuits from the sensor and motor information induced by SMA; we called this stage, the *passive stage*. In the second stage, we manually identify a set of gains that scale the reflex networks by trial-and-error, such that we can achieve coordinated behaviour. In contrast to the previous stage, we called this stage, the *active stage*.

**Figure 2:**
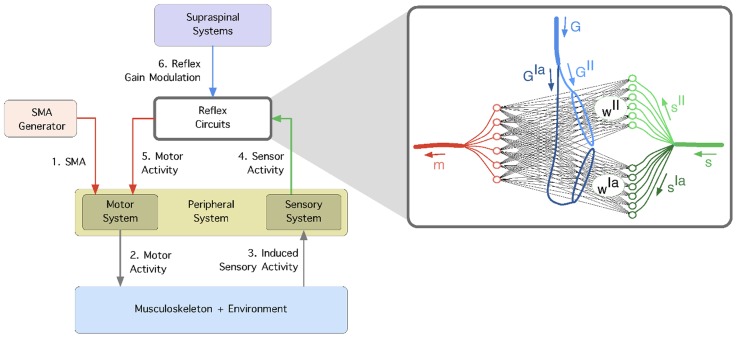
The learning framework. 1) Spontaneous motor activity stimulates the motor system and 2) causes the muscles to contract. 3) The generated forces are propagated through the musculoskeletal system (and the environment) and induce sensor stimulation in the primary (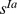) and secondary (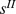) spindle afferent fibers. 4) The correlation between the sensor and motor signals is used to self-organize the reflex networks, 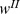 and 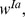 which mediate the connectivity of afferents 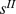 and 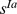 respectively. 5) The reflex circuits are modulated from supraspinal systems using gains 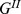 and 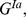 which independently scale the reflex networks 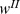 and 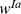 respectively.

The information flow in our developmental model is shown in Figure 2; the passive stage involves steps 1–5 and the active stage involves step 6. The model can be summarised as follows. First, spontaneous motor activity generates muscle contractions in the form of muscle twitches. Second, these twitches produce muscle forces which are propagated through the musculoskeletal system (and the environment). Third, changes in the musculoskeletal state induce sensory information, which fourth activate various sensory receptors [26]. Fifth, the correlation between the sensor and motor signals determines the pattern of connectivity of the different reflex circuits [29], [30]. Sixth, once the reflex circuits are in place, their strength is modulated by supraspinal systems to achieve coordinated behaviour. In the following sections, we describe in detail each of the sub-models used as well as the experimental methods underlying our experiments; we will start with the musculoskeletal and the environment systems.

### Model of musculoskeletal and environment systems

**Figure 3:**
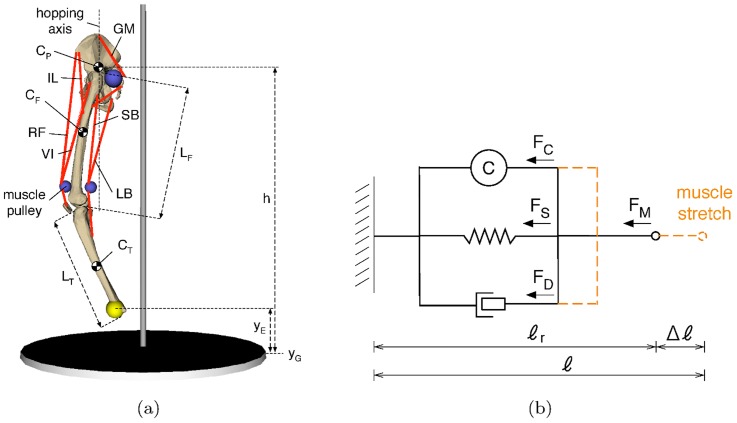
The default musculoskeletal model used in our experiments. a) The leg model comprises six muscles, the iliacus (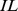), the rectus femoris (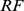), the vastus intermedius (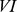), the gluteus maximus (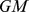), the long biceps (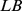), and the short biceps (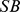); 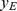 and 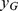 represent the height of the end-effector and the ground respectively, and 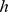 represents the height of the hip. 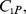 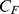 and 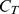 show the centers of mass of the pelvis, femur and tibia, respectively. 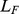 and 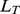 are the lengths of the femur and the tibia, respectively; the centers of mass of these bodies are located in the geometrical center of the body. b) The 3-element muscle model used; it consists of a spring (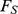) and a damper (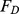) in parallel to the contractile element (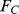).

The musculoskeletal system consists of a leg model comprising three rigid segments: pelvis, femur and tibia (see Figure 3a). The model is implemented in MATLAB SimMechanics and visualized using the 3D Animation Toolbox (also from MATLAB). The system is actuated primarily by six muscles, but in one of the experiments we use a four-muscle configuration (see Results). The masses of the rigid segments are set to 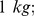 the lengths of the femur, 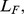 and tibia 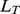 are set to 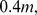 which is their approximate length in a human with 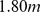 [31] p.302. The hip and knee joints are simulated as revolute joints. An additional joint is added to the hip in order to restrict the movement of the pelvis to a vertical motion. This joint also prevents the rotation of the pelvis. We call this prismatic constraint, the *hopping axis* (see Figure3a). It is worth mentioning that our model is intrinsically a 3D model, in the sense that every point in it (e.g. the joint locations as well as the attachment points of the muscles) is defined by three coordinates. However, in practice, given that the hip and knee joints are both hinge joints aligned along a single plane, the motions of all the rigid bodies are restricted to 2D movements.

The muscle model used is a variation of the 3-element Hill-muscle model [32], [33], in which the tendon is simulated as a rigid element (see Figure 3b). This simplification offers significantly higher computational speed at the expense of a relatively small error in accuracy [34] (see Discussion). The model consists of a contractile element placed in parallel to a spring and damper systems. The total force, 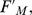 produced by a given muscle is given by:
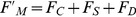
(1)where 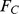 is the force produced by the contractile element, 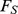 is the force produced by the parallel elastic element, and 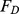 is the force produced by the parallel damping element. 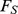 and 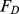 are given by:
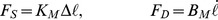
(2)where 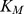 is the spring constant of the parallel elastic element, 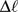 is the muscle deformation given by the difference between the current length, 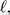 and the resting length, 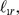 of the muscle, 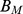 is the damping constant of the muscle, and 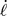 is the rate of change of the muscle length. In most of our experiments we simply set 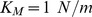 and 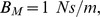 but we also present results with biologically plausible parameters (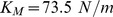 and 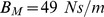) as identified in [35]. The results obtained show that the model produces consistent results for different variations of these parameters (see Results). The active force, 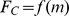 is proportional to the activation 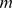 of the muscle. The activation of each muscle is low-pass filtered to prevent large and instantaneous force variations in the muscle. The filter uses a time constant of 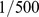 and a passband gain of 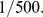


The asymmetric conditioning of the muscles (i.e. the fact that muscles can only produce contractile, but not extension, forces) was simulated by the following equation:
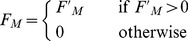
(3)


The ground contact model, which simulates the reaction forces produced when the end-effector is in contact with the ground, is computed as a spring-damper system:
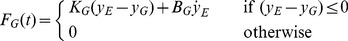
(4)where 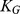 and 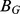 are the spring and damping coefficients of the ground, respectively, 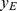 is the position of the end-effector on the vertical axis, and 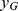 is the location of the ground also on the vertical axis (see also Figure 3a). By default these parameters are set to 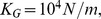 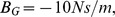 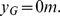 We have varied these parameters to the extent that the contact with the ground looks realistic and obtained similar results to those reported here (see Results).

### Model of the peripheral system

The peripheral system is given by the sensor inputs from, and the motor outputs to, our leg model. On the motor side, we use the muscle activation 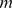 which defines the force produced by the contractile element of the muscle. We denote 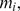 the motor signal of muscle 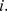 On the sensor side, we use approximations to the primary and secondary afferent fibres as observed in biological muscle spindles. In general, we use muscle velocity to approximate the response of primary fibres and muscle length to the approximate the response of secondary fibres. More specifically, the primary afferents are modelled as the rate of change in muscle length 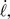 and the secondary afferents as the muscle deformation 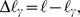 where 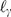 provides a reference muscle length (see Figure 3b). Note that parameter 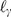 is different from 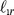 which is part of the muscle model; whereas the latter is the mechanical resting length of the muscle the former provides a way to set the desired length of the muscle. The parameter 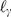 is a simplification of fusimotor interactions between γ- and *β*-motoneurons, which act on the nuclear bag fibres to modulate the sensitivity of the spindle receptors. It can be seen as having a similar effect to that of 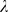 in the TCT [15]. For each muscle, this reference value is obtained by manually setting the hopping posture of the leg and recording the muscle length. We have tested different leg postures and, as far the leg is kept in natural alignment for hopping (i.e. with a slight flexion of the hip and knee), we could reproduce the results presented here. We denote the primary and secondary sensory afferents from a given muscle 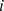 as 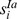 and 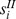 respectively. When referring to a general sensor input from muscle 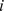 irrespective of its type, we simply use 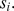


### Model of spontaneous motor activity

The model of spontaneous motor activity (SMA), which is carried out during the passive stage, simulates the production of muscle twitches observed during early foetal development [2], [3], [36] as well as during sleep throughout the mammalian life span [37], [38]. This type of activity causes the *α*-motoneurons to fire spontaneously, which in turn produces muscle contractions independently from sensory stimulation.

The model of SMA used in this paper is intended to portray the process of myoclonic twitches observed during REM sleep, many of which “are dominated by a single muscle” [29] (see Discussion). It is carried out by contracting all muscles in sequence; each muscle is contracted ten times allowing 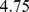 seconds between each twitch for the system to recover and stop oscillating. To mimick closer the environmental conditions in which SMA occurs, we disabled gravity during the reflex learning stage. The reason for this is two-fold. First, during sleep the body is typically displaced horizontally, and thus the influence of gravity on the sagital plane of the body is minimal. Second, in uterus the boyance provided by the uterine environment also reduces substancially the effects of gravity. Note thought that, as shown in [39], this is not a requirement of the system.

The sequence of muscle contractions during SMA is 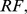 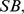 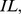 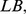 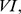 and 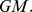 Throughout the entire period of SMA (i.e. the passive stage), the patterns of muscle contractions are fixed and do not change over time, i.e. they are carried out in a purely feedforward way and are thus unaffected by either sensor activity or reflex circuits. This is consistent with observations of SMA triggered in the context of REM sleep, during which reflex circuits seem to be inhibited [40]. For a twitch in muscle 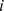 we set 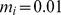 for 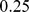 seconds. A sequential activation is chosen for practical reasons; the assumptions and limitations of the model are discussed at length in the Discussion section.

### Model of reflex circuits and supraspinal system

The self-organization of the reflexes is carried out by using the differential anti-Oja rule similarly to [29], [30] (see also [41]). This rule is a normalized version of the anti-Hebbian rule [42], which in turn consists of the additive symmetric of the well-established Hebbian rule [43] (see also [44]). Using the anti-Oja rule, the change in the reflex connection strength at time 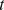 is given by:
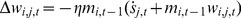
(5)where 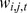 is the connection between the motor element of muscle 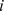 and receptor 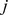 at time 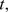 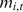 is the motor activity of muscle 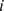 at time 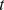, 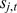 is the sensor activity of receptor 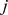 at time 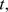 and 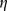 is the learning rate. In our system we set 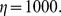


Once the reflex circuits are established, the motor signals generated in response to stimulation of the sensor receptors 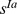 and 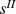 are as follows:
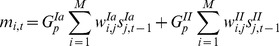
(6)where 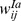 is the connection between sensor receptor 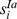 and motor 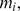 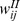 is the connection between sensor receptor 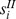 and motor 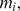 and gains 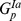 and 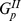 are global network parameters which modulate the overall strength of the reflex networks (involving 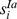 and 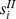 afferents, respectively) during the movement phase 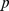 (for gain modulation see [45]–[47]). The delay between sensor and motor activity is given by one simulator timestep (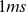) which is consistent with that of short-latency reflexes like those presented here [48], [49]. All the experiments described in this paper use a single set of gains to produce the coordinated behaviour (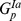 and 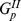); for simplicity we will simply denote these gains as 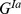 and 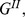 respectively. The only exception are the experiments carried out to address the hypothesis that behavioural transitions can be achieved by changing the reflex gains over time (see Results). In these experiments, we apply a different set of reflex gains for different phases of the coordinated behaviour; for example, we apply a given set of gains during the stance phase and a different set during the flight phase.

### Experimental procedure

All the experiments follow a similar protocol in which reflex circuits are self-organized during the passive stage, and are then modulated in the the active stage. In the passive stage we produce 10 twitches in each muscle sequentially. The sequence of muscle activations is: RF, GM, IL, LB, VI, SB. Modifying this order produces only marginal changes in the reflex matrices obtained (see also Discussion). At each simulation step, the reflex circuits are updated according to eq.5. The initial connection weights of the networks, 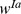 and 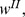 are set to zero. In the active stage, we set the gain parameters 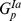 and 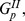 we drop the leg from a height of 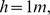 and evaluate the hopping pattern obtained against the two criteria described below.

It is not the goal of this paper to use feedback control of the hopping height, i.e. we do not have an explicit reference height that is intended to be achieved by the leg. Our goal is to show that once the reflexes are in place, the modulation of the reflex networks is sufficient to coordinate the muscle activity to achieve a stable hopping pattern. In our experiments, we tuned the reflex gains 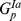 and 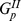 manually. This is a relatively simple task which resembles that of tuning a *PD*-controller (see Discussion). To measure the quality of the hopping behaviour we use two criteria that have to be met; we call these criteria hopping stability and conservation of the hopping height. The hopping stability, 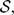 measures the average difference of the hopping height achieved in two consecutive hops, and it is given by:
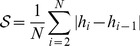
(7)where 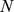 is the total number of hops and 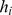 is the peak height achieved at hop 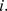 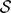 is measured in millimetres per hop. A stable hopping pattern is given by a low 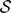. We set 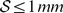 to be a stable hopping pattern, which indicates that on average the difference between two successive hops should be less than (or equal to) 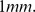 In addition, the hopping behaviour is also considered unstable if it achieves at least one hopping peak outside of the boundary 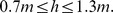


The conservation of the hopping height, 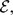 is given by the slope of the line fitted over the peaks of 100 successive hops, and it is measured in millimetres. In the long run, for 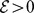 the hopping height is increasing, for 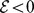 the hopping height is decreasing, and for 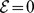 the hopping height is kept at a stable value. We consider a system where 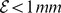 to be able to conserve the hopping height.

## Results

The results obtained with our developmental model are reported below. In the first section we use the default leg model (show in Figure 3) to address the hypothesis that SMA can propel the self-organization of reflex circuits. In the second section we show that once these circuits are established, they can be modulated to achieve coordinated behaviour. In the third section, we modify the default leg model in several ways to address the hypothesis that our developmental model can naturally cope with morphological changes. In the fourth section, we show that we can switch between different coordinated behaviours simply by modifying the reflex gains over time. Finally in the fifth section, we show how our model scales to produce simple point-to-point trajectories.

### Self-organization of reflexes from SMA

**Figure 4:**
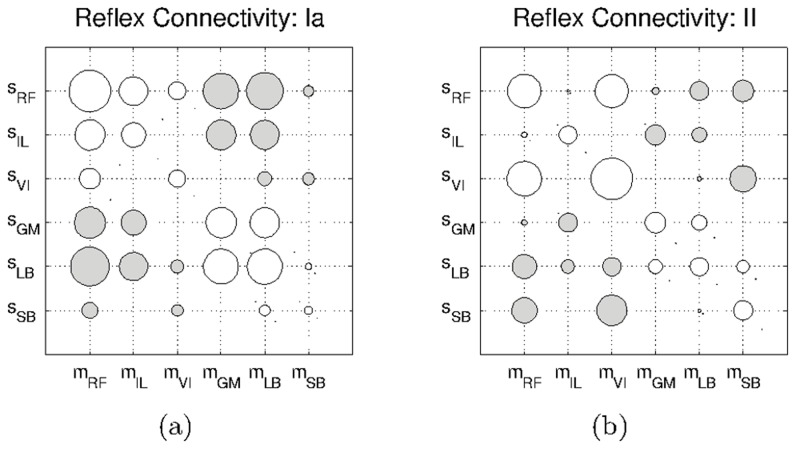
Hinton diagrams of the reflex circuits obtained with the default leg model. a) Circuits obtained for the *Ia*-type afferents, and b) those obtained for the *II*-type afferents. Unfilled circles represent excitatory connections, and filled circles represent inhibitory connections.

We first address the hypothesis that meaningful reflex circuits can be self-organized from SMA. The experiment is carried out by triggering ten twitches in each muscle of the default leg model and by correlating the resulting sensor and motor activity during the passive stage. The reflex circuits obtained are shown in Figure 4. These circuits were obtained using a twitch amplitude of 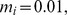 which produces almost unoticeable muscle contractions. In Figure S1 we show the reflex circuits obtained with an activation of 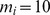 (see Movie S1). The figures show connectivity matrices in which each element represents a connection between a sensor and a motor elements. Unfilled circles represent excitatory connections, filled circles represent inhibitory connections; the size of the circle represents the magnitude of the connection.

From a qualitative point of view the circuits generated are similar in the two sensor modalities. We observe excitatory connections between motor elements and homonymous type-*Ia* and type-*II* afferents (e.g. 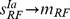 and 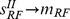 respectively). The same type of connectivity is obtained for synergist interactions (e.g. 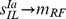 and 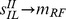). This connectivity is consistent with the myotatic reflex circuitry. Antagonist interactions are mediated by inhibitory connections (e.g. 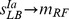 and 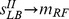). This circuitry is consistent with the reciprocal inhibition reflex. Together the myotatic and the reciprocal inhibition reflexes form the stretch reflex, which is one of the best known circuits in the mammalian spinal cord (see Discussion). The similarity between the two reflex networks is not surprising since the general trend in both sensor inputs is very similar within a given twitch. From a quantitative point of view the differences observed are associated with the inherent difference between the two physical quantities that each receptor measures: positional information for the type-*II* sensors and velocity information for the type-*Ia* sensors.

In addition, we observe that some of the connections obtained present very small magnitudes. This can be observed for example for the connection between 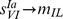 (or for the reciprocal connection between 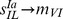), the magnitude of which is too small to appear in the figure. The reason for this is that these connections mediate sensory and motor elements located at different joints. This can clearly be seen in the case of the Iliacus (which actuates the hip); when a twitch occurs in the Iliacus, it induces significantly less sensor activity in the muscles around knee (e.g. 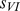 or 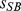) than in those around the hip (e.g. 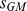 or 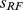). This reduced sensory activity results in connections with smaller magnitudes.

**Figure 5:**
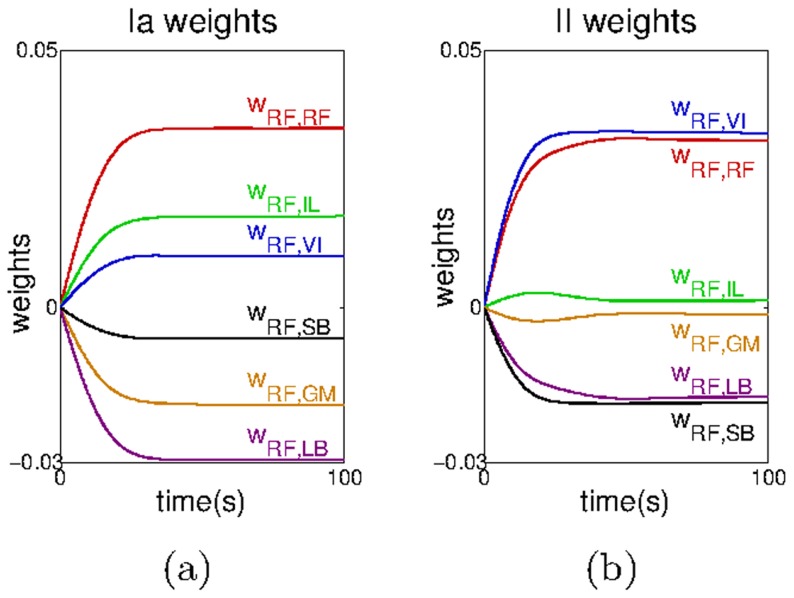
Convergence of all the reflex weights involving the Rectus Femoris motor element, 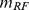. a) Reflex weights relative to the *Ia* -type afferents, and b) relative to the *II*-type afferents. For clarity, the raw data has been smoothed using a moving average filter with a window of 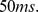

In Figure 5, we show how the connections between the motor element of the Rectus Femoris, 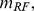 and the sensor afferents of all the muscles, 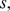 evolve during the passive stage. As can be seen the weights converge to rather stable values after around 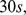 time at which each muscle has twitched once. When modifying the 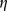 parameters in eq.5 our observations are consistent with those in the literature; lower values of 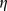 (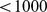) lead to a slower convergence of the weights, and values significantly higher (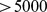) lead the weights to oscillate without any real convergence. We observe a similar convergence for the other muscles. These results indicate that spinal reflex circuits can be obtained from the self-organization of sensory and motor information induced by SMA.

### Coordinated behaviour from the modulation of reflexes

To address the hypothesis that coordinated behaviour can be achieved from the self-organized reflex circuits obtained in the previous section, we drop the leg from a height and search manually for a set of reflex gains (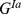 and 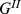) that can make the leg hop in a stable pattern (see Models).

**Figure 6:**
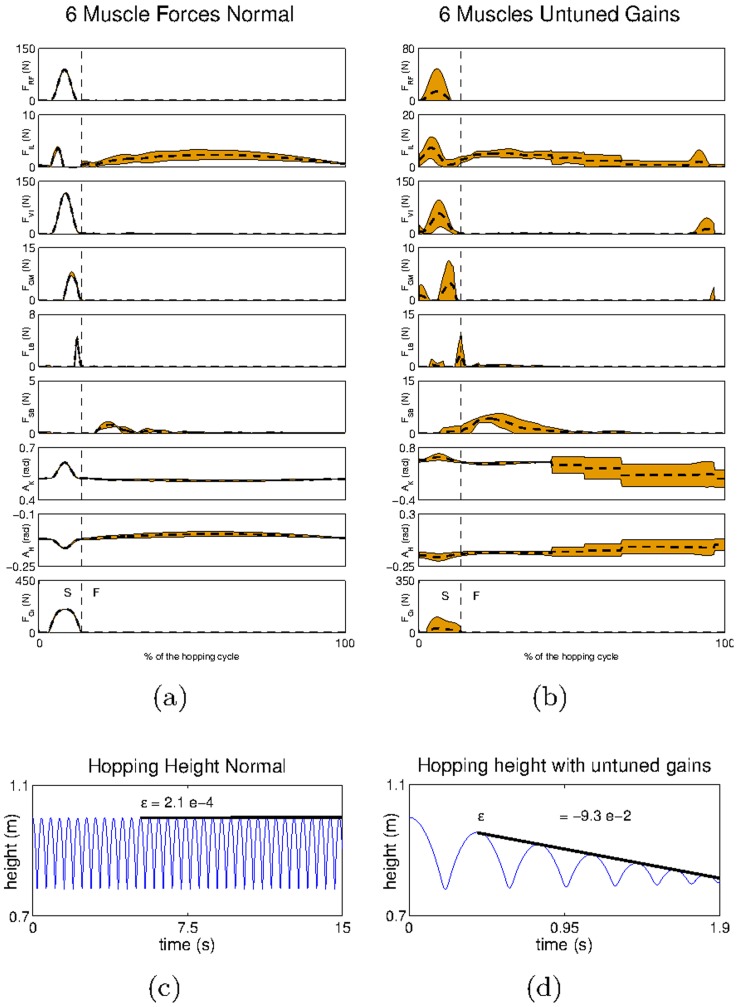
The hip trajectory and the mean and standard deviation of the kinematic and dynamic variables obtained for the default leg model. Kinematic and dynamic variables obtained for the system with a) an appropriate set of gains, and b) an inappropriate set of gains. S refers to the stance phase (when the end effector is in touch with the ground) and F refers to the flight phase (when the end effector is in the air). In b, because each hop has a different duration, the data relative to each hop has been linearly interpolated to match the durations across different hops. Note that in this plot the time indicated for the stance-to-flight transition is only relative to the first hop. In subsequent hops, and because they progressively decrease in duration, this transition occurs earlier than illustrated by the marker. The hip trajectory recorded for the system with c) an appropriate set of gains, and d) an inappropriate set of gains.

Figure 6a shows the mean and five times the standard deviation of the main kinematic and dynamic variables collected after 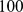 hops carried out with an appropriate set of gains (see also Movie S1.IV). These variables are the muscle forces, the hip and knee angles and the ground force (see Figure S2 for similar results achieved using the reflex circuits obtained with a twitching amplitude of 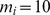). All the variables have been aligned with respect to initial contact with the ground (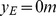). As can be seen, the standard deviation of each parameter is relatively low, demonstrating that the hopping pattern is stable (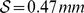). We can also see that the hopping height is conserved as indicated by the low value of 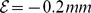 (Figure 6c).

To investigate the sensitivity of the results with respect to the ground model used and the weight of the leg we carried out two additional experiments. In the first, we test the system with three different ground models: ground model 1 (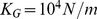 and 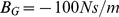), ground model 2 (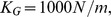 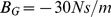) and ground model 3 (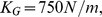 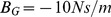). In the second we test the system with a leg with the double of the weight of that of the default model (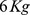 in total). The results obtained are consistent with those presented for the default leg model; they can be observed in the supplementary materials, in Figures S3,S4.

When analysing the muscle activity produced shortly after the touch down, one can observe a force increase in the extensor muscles 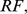 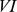 and 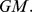 At this stage all these muscles undergo a sudden extension, which increases the activity of their corresponding 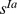 and 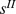 sensor receptors; this increased activity, in turn propels the reflex circuits to contract these muscles. A small force is also observed in the 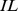 (which is a flexor muscle); this force is mainly caused by the stretch of the synergist 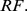 When the leg leaves the ground, the situation is inverted: the flexors muscles (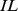 and 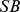) are now those undergoing a stretch as the leg over-extends beyond the desired posture. This causes the reflex circuits to increase the activity of these muscles, which brings the leg to its natural pose and prepares it for the next hop. Both before and after touch down the torques produced at a joint are distributed across the meaningful muscles (see also [50]). This is what we mean by *coordinated behaviour*; the appropriate muscles are recruited in a timely way such that the final behaviour is attainable.

**Figure 7:**
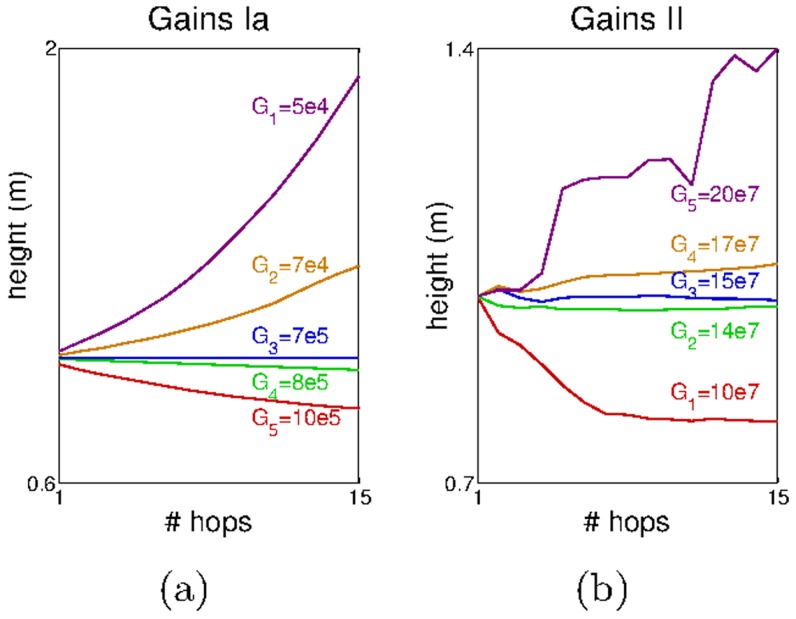
The hopping height progression achieved for different a) 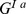 and b) 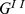. The large magnitude of the gains is justified by the fact that we use SI units in the afferents – 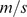 for type-*Ia* afferents and 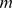 for type-*II* afferents.

To illustrate the importance of the gain parameter tuning process we show in Figure 6b the results obtained with parameters 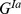 and 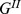 smaller than those used in Figure 6a (see also Movie S1.II-III). With the new gains, the system is clearly unstable (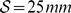) and it does not fulfil the conservation of the hopping height criterion (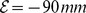) as we observe a regular decrease in the hopping height (Figure 6d). Moreover this is accompanied by a clear increase in the standard deviation in all the muscle (and ground) forces as well as in the hip and knee angles (Figure 6b). A more in-depth analysis of the effect of each gain on the hopping stability is shown in Figure 7. In this experiment we varied each of the gain parameters and observed the progression of the hopping height. For each plot we modified one of the gains while keeping the other fixed at the value that produced the stable hopping pattern shown in Figure 6b. In Figure 7a we varied 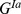 and in Figure 7b we varied 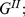 in each plot 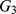 represents the value of the respective gain that achieved the stable hopping pattern.

The results show that around the value 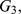 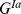 is inversely proportional to the hopping height, i.e. the smaller the gain the higher the hopping height (Figure 7a). This is because large values of 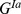 reduce the duration of the stance phase, and prevent the leg to markedly change its posture. Consequently, this reduces the deformation in the extensor muscles (after the contact with the ground) and decreases the forces that would otherwise be produced by the reflex network modulated by 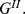 In contrast, the higher the value of the gain 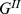 the higher the hopping height achieved (Figure 7b). This result is expected since by increasing 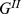 we are increasing the forces produced in response to a given deformation, without altering the forces produced by the 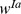 reflex network.

Next, we addressed the hypothesis that coordinated behaviour can only occur once meaningful reflex circuits are in place, e.g. reflex circuits that reflect the interactions in the musculoskeletal system. In practice, it could happen that many different reflex circuits, if provided with the right gains, could lead to stable hopping, in a way reminiscent of reservoir computing, where a non-linear recurrent network with the connections weights selected from random distribution, can be shown to produce outputs that can be linearly combined to achieve a desired function [51], [52]. We have tested more than thirty randomly generated reflex circuits and we have not managed to find appropriate gains to achieve any resemblance to a single hop. The resulting systems mostly fall onto the ground without being able to produce a single hop. These results show evidence that our framework establishes appropriate relations across the different muscles such that a stable hopping pattern can be obtained.

**Figure 8:**
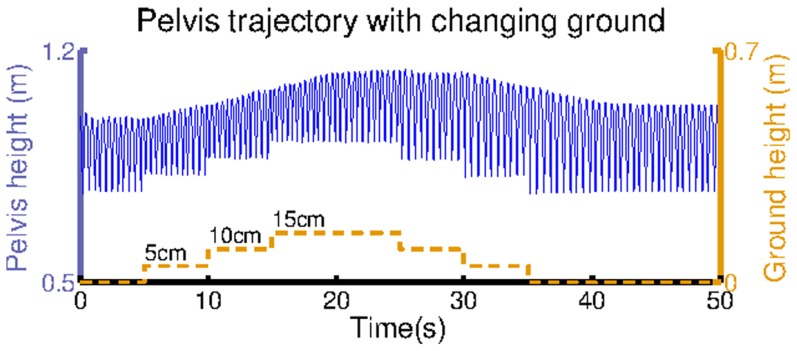
The hopping height achieved when modifying the height of the ground. The hopping height is shown in blue and the ground height is shown in orange.

To show that the hopping stability obtained is not restricted to the specific hopping height from which the leg is dropped we perform an experiment in which we dynamically change the ground position. In this experiment, while the leg is hopping we increase the ground height from 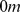 to 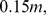 in 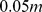 intervals, and decrease it back to 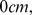 also in 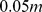 intervals. Our results are shown in Figure 8. In spite of the varying ground position the leg keeps hopping, which shows the capability of the model to cope with external perturbations (see also Movie S1.V).

### Adaptivity to morphological changes

To address the hypothesis that our system can naturally adapt to changes in the body morphology, we systematically modified the default musculoskeletal system (see Figure 3a), and analysed how the developmental model adapts to the changes while maintaining the coordinated locomotor behaviour. We made three different types of changes. First, we removed the two bi-articular muscles (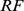 and 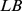), leaving the leg only with four muscles. Second, we modified the attachment points of the 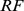 such that the muscle is longer while maintaining the physical connection at the hip and knee. Third, we modify the 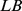 such that the muscle has the same geometrical path as the 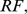 i.e. the two muscles are placed in parallel and have the same mechanical effects at both the hip and the knee.

In the adaptation experiments, we set the initial reflex circuits to be those identified for the default leg model (see Figure 3a). The experiments start with a passive stage, in which the initial reflex circuits are updated according to the new musculoskeletal configuration; and then continue with an active stage, in which the updated reflex circuits are modulated to achieve a stable hopping pattern, as in the previous sections.

**Figure 9:**
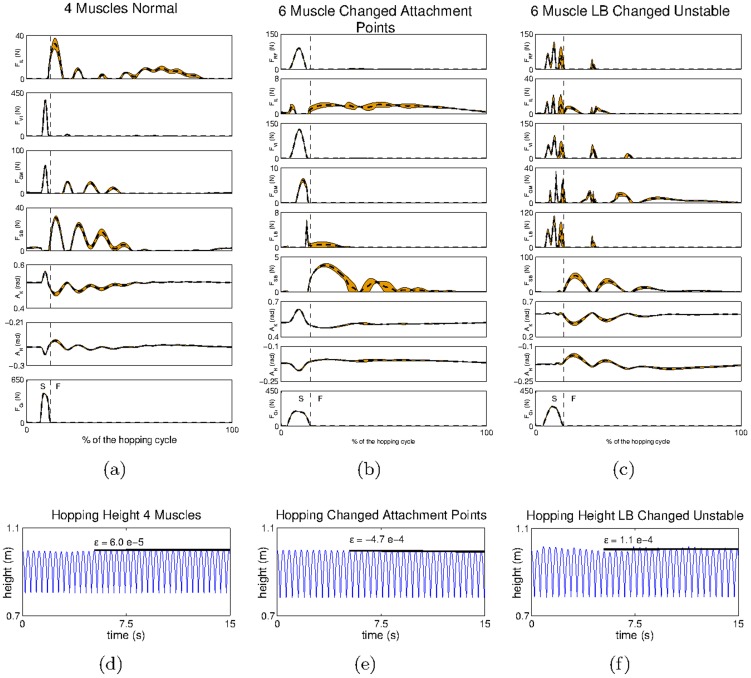
The hip trajectory and the mean and standard deviation of the kinematic and dynamic variables obtained for the modified leg models. Kinematic and dynamic variables obtained for the system with a) four muscles, b) modified 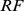 and c) misplaced 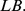 S refers to the stance phase (when the end effector is in touch with the ground) and F refers to the flight phase (when the end effector is in the air). The hip trajectory recorded for the system with d) four muscles, e) modified 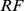 and f) misplaced 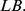

With respect to the removal of bi-articular muscles, the reflex weights obtained are similar to those in Figure 4, taking into account that any connections involving sensor or motor elements of the 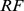 or 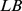 are absent (given that these muscles do not exist in this modified leg model). The hopping behaviour achieved with this system is shown in Figure 9a,d. In spite of the larger oscillations in the muscle forces and in the joint angles observed during the flight phase of the movement, the system manages to achieve a stable hopping pattern (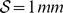) and to conserve the hopping height (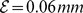) (Figure 9d). The larger oscillations in the muscle forces and the joint angles achieved by this system, when compared to those in the default system, are consistent with mechanical observations described in [53], [54], where the stabilizing role of bi-articular muscles has been put forward.

Relative to the changes in the attachment points of the 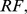 we obtain qualitatively similar reflex connectivity as that in Figure 4 (not shown); we observe only minor quantitative differences in connections involving the sensor and motor elements of the 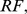 which are due to changes in the attachment points of the modified system. The hopping behaviour obtained is shown in Figure 9b,e. The profiles of muscle forces and joint angles obtained are relatively similar to those observed with respect to the default leg model (shown in Figure 9a,c). Our results confirm our intuition; given that the two mechanical systems are not fundamentally different the results obtained are very similar, both in terms of reflex circuitry and of behavioural coordination (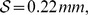 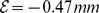).

**Figure 10:**
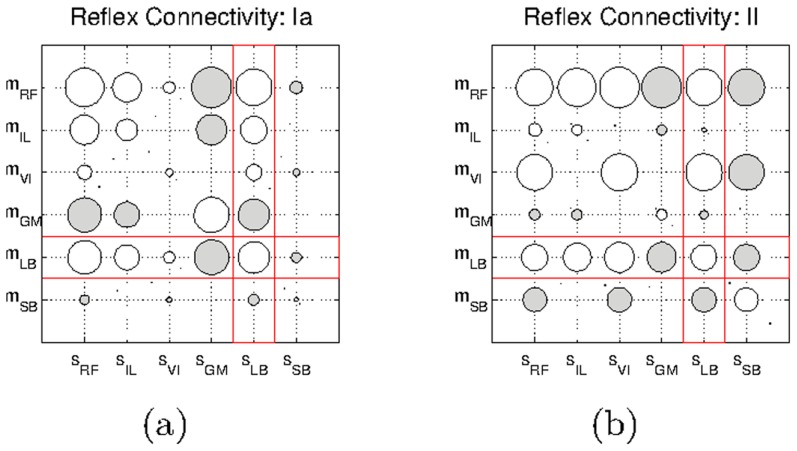
Hinton diagrams of the reflex circuits obtained for the system with the modified 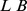. a) Connections involving *Ia*-type afferents and b) *II*-type afferents. Unfilled circles represent excitatory connections, and filled circles represent inhibitory connections. The red squares highlight the modified connections with respect to the default system.

The reflex circuits resulting from the modification of the geometrical path of the 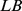 are shown in Figure 10. When compared with the circuits obtained for the default configuration (see Figure 4), one can observe that all the connections with the 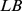 afferents (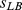) and motor elements (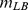) have been drastically altered (marked in red); the only exceptions are the homonymous connections (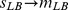). In fact, these connections are now very similar to those of the 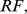 which reflects the identical geometrical path followed by both muscles in the new leg configuration.

**Figure 11:**
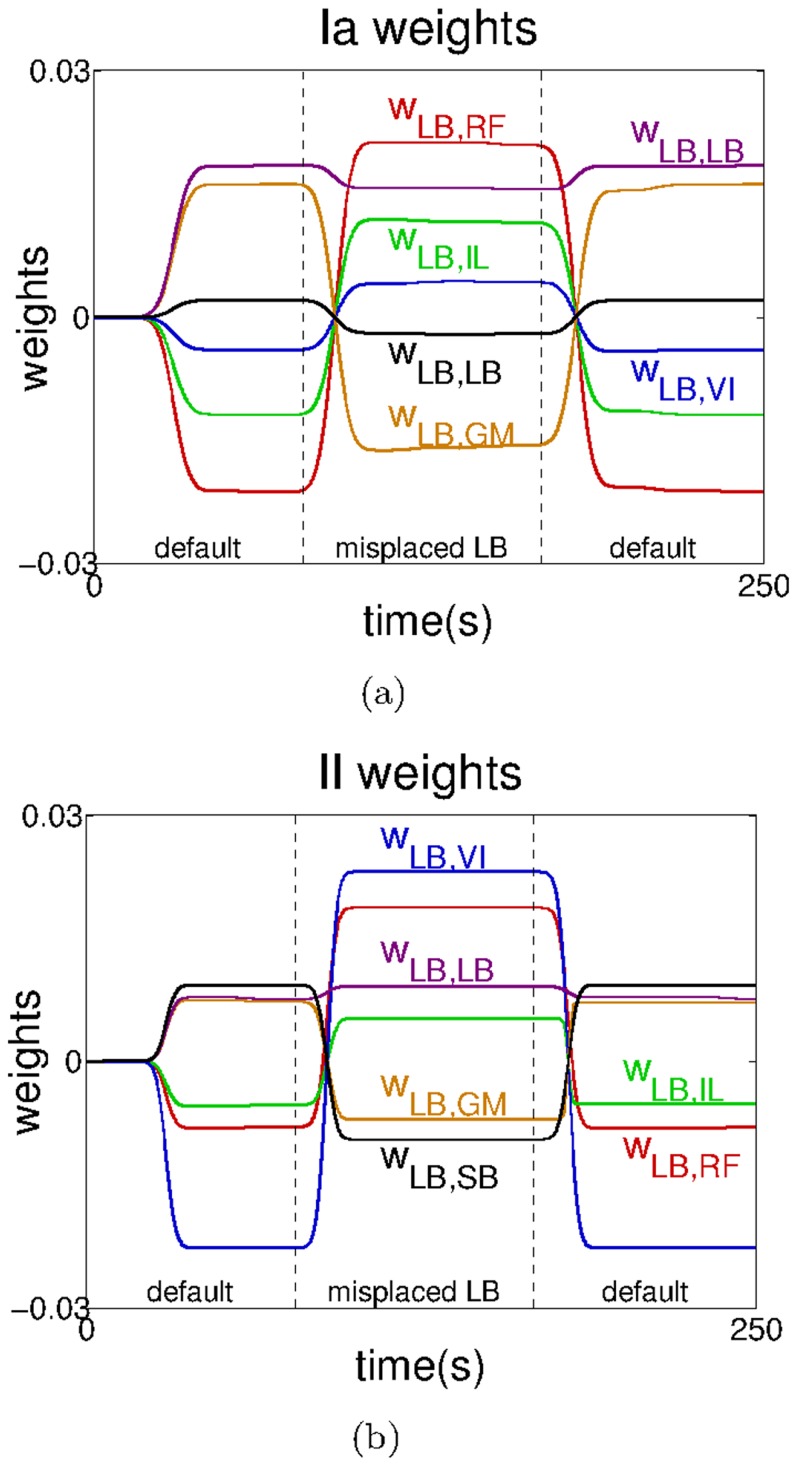
Changes in the reflex weights of the 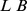 motor element (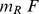) when passing from the default system to the system with the misplaced 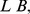 and back to the default system. Connections involving a) the *Ia*-type and b) the *II*-type afferents. For clarity, the data has been smoothed using a moving average filter with a window of 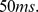

In Figure 11 we show the progression of all the connections involving the 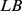 motor element 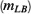 when switching from the default system, to the system with the misplaced 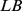, and back to the default system. When the transition to the system with the misplaced 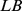 occurs, all the connections change signs with the exception of the homonymous connection. Similarly, when changing back to the default system, the connections converge to the values they initially had, presenting a clear case of reflex adaptation.

Figure 9c,f, shows the hopping pattern obtained for the system with misplaced 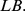 In the new configuration, although the system is almost capable of conserving the hopping height (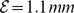 in Figure 9f), it cannot achieve a stable hopping pattern (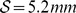). The reason for this is that the modified system is rather unbalanced; it has three hip flexors (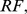 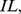 and modified-*LB*) acting against a single hip extensor (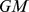), and three knee extensors (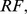 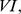 and modified-*LB*) acting against a single knee flexor (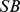). Because all the muscle activities are controlled using only two parameters (through the reflex circuits) it is difficult to find a set of parameters that can balance the torques at the joints both during the stance phase (which requires mostly the activation of extensor muscles) and the flight phase (which requires mostly the activation of flexor muscles). Using the connectivity matrix obtained for the default system in the modified system, did not result in any behaviour resembling hopping, reinforcing the idea that the symbiosis between reflex matrix and body morphology is an essential element of the model. Furthermore, and consistent with our results for the 4-muscle arrangement, we observe larger oscillations in the muscle forces during the hopping cycle, which reinforces the idea that bi-articular muscles (which in the new configuration are absent in the posterior part of the leg) are an important stabilizing mechanism.

### Changing the modulation gains over time

In the experiments described so far, we observed that we could regulate the hopping behaviour using only a set of gains (i.e. 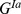 and 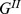). This was surprising to us since the two phases of hopping (stance and flight phases) have very different requirements in terms of muscle forces. During the stance phase hip flexors and knee extensors are required to pump energy into the system to overcome any energy losses resulting from the contact with the ground, whereas during the flight phase, hip extensors and knee flexors are required to bring the leg as close as possible to its initial posture, which prepares the leg for the next hop. This issue became clearly apparent in the last experiment of the previous section (see Figure 9c,f), in which we had difficulties in balancing the two requirements using only two parameters.

A more general way of using the reflex gains, is by exploiting the full potential of eq.6 and set the gain parameters differently during the stance phase and the flight phase of the hopping behaviour. Indeed, there is plenty of evidence that this is the case with several mammalian coordinated behaviours [7], [55]–[57]. Although such a system requires the control of more gains (in our case, two gains over time instead of two fixed gains) their identification is an easier task, because one can clearly partition the objectives of each phase of the movement. In the case of hopping, during the stance phase, we set the gains such that the leg can jump a certain height, without the constraint that the same parameters will be capable of bringing the leg to its desired posture during the flight phase. And conversely, during the flight phase we set the gains such that the leg can recover its posture, without the constraint that the same gains are able to make the leg jump during the stance phase.

The experiments carried out here are similar to those in the previous section, the only difference being that instead of using a single set of gains we use two different sets, one for each hopping phase. During the flight phase we set a pair of gains 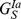 and 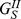 such that the leg can jump to a height of 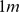 and during the flight phase we set gains 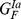 and 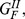 such that the leg can recover its original posture. As in the previous experiments the gains were tuned manually. The two phases are differentiated by whether or not the leg is in contact with the ground (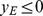); in the biological system this information could be retrieved from tactile sensors at the end-effector as shown in [58].

We first demonstrate the results of the new strategy using biologically plausible muscle parameters (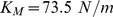 and 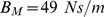). When using this model with only two gains, we could achieve a hopping pattern that was close to stable, but that did not meet our tight stability criteria. However, with four gains we can clearly achieve a very stable hopping pattern (see Figure S5).

**Figure 12:**
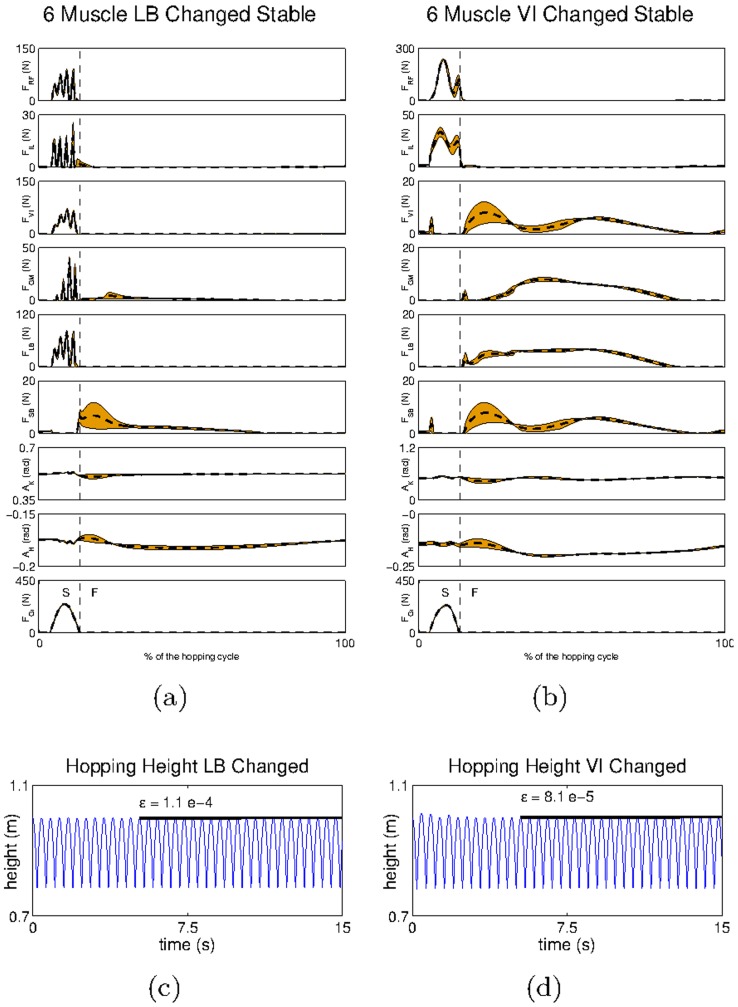
The hip trajectory and the mean and standard deviation of the kinematic and dynamic variables obtained for the default modified models using dynamic gain modulation. Kinematic and dynamic variables obtained for the system with a) misplaced 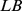 and b) misplaced 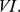 S refers to the stance phase (when the end effector is in touch with the ground) and F refers to the flight phase (when the end effector is in the air). The hip trajectory recorded for the system with c) misplaced 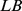 and d) misplaced 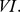

Next, the importance of the gain tuning is examined through the case study, which was not fully successful in the previous section, i.e. the one with the modified 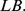 In Figure 12a,c one can observe the hopping pattern obtained in this system using one set of parameters for each movement phase. Using the new strategy, one can still observe oscillations during the hopping cycle due to the lack of a bi-articular muscle on the posterior part of the leg (Figure 12a). However, when analysing the hopping height achieved we can observe that it is now stable across different hops (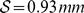 and 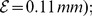 this contrasts with Figure 9f in which the hopping height changed considerably from one hop to the next 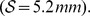 To show the generality of our results we show an additional modification of the mechanical system, in which the 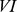 is placed parallel to the 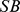 (see also Movie S1.VI). Similarly to the modification of the 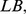 we could not obtain a stable hopping pattern for this system with a single set of gains. Our results are shown in Figure 12b,d (see also Movie S1.VI). With the exception of the muscle oscillations, which are considerably reduced in this system, we obtained very similar results when compared to the system with the modified 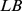 (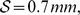 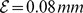).

We then tested the modified systems used in Figure 12, with the reflex circuits learned for the default system. In these experiments the reflex networks do not fully capture the interactions of the musculoskeletal system, in particular the relations with the 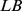 and the 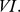 In both modified systems we could only make the leg hop a few times, but could not find a set of gains, 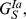 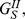 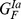 and 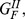 that achieved a stable hopping pattern. Critically, during the flight phase the hip and knee angles diverged progressively from those dictated by the desired posture, until the leg reached a posture which prevents it from jumping. These results reinforce the importance of the coupling between reflex circuits and body morphology, and indicate that, at least in our model, appropriate reflex circuits might be necessary to achieve coordinated behaviour.

**Figure 13:**
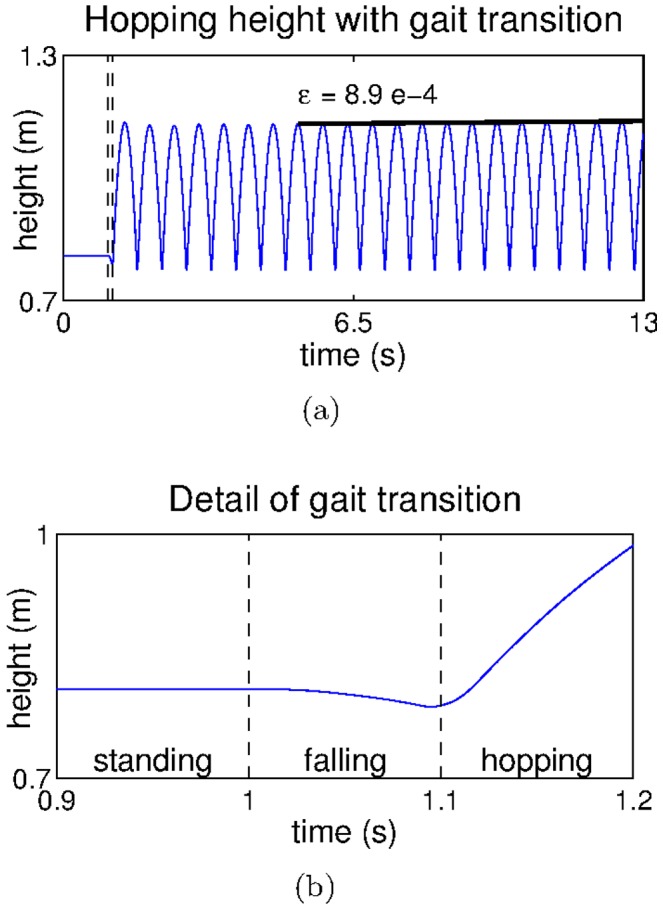
Hopping transitions. a) The hopping height achieved after the behavioural transition and b) detail of *a* showing the behavioural transition.

Another important aspect of the dynamic tuning of gains can be found in the transitions between different behavioural patterns. To test the hypothesis that behavioural transitions can be obtained by changing the modulation gains over time we carried out one additional experiment. In this experiment we show the switching from a standing behaviour, which keeps the leg standing on the ground, to the dynamic hopping behaviour described in the previous sections. We start the experiment by identifying three pairs of gains: one pair that allowed the leg to stand on the ground holding its own weight, another pair that caused the leg to fall down (basically, 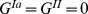), and a final pair of gains that produced the hopping behaviour (here, we use the same gains identified in the context of hopping with the default leg model in Figure 6a,c). We then set these gains sequentially; first we set the standing gains, then we set the falling gains for 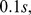 causing the leg to start falling, and finally we set the hopping gains. Our results are shown in Figure 13 (see also Movie S1.VII). As can be seen, the change in the gain parameters is sufficient for the system to achieve stable hopping starting from a standing position.

When investigating the sensitivity of the system with respect to the falling time, we observed that using the same gains we could still hop when setting the falling time to 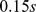 but not when it was set to 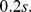 However, if we change the hopping gains, we can still obtain a stable hopping pattern for a falling time of 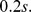 Overall the results of this section show that our model can be generalized to achieve behavioural transitions by changing the modulation gains over time.

### Scalability to point-to-point trajectories

The experiments described so far have shown how the system can achieve a stable rhythmic hopping behaviour by modulating the gain parameters 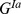 and 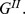 Modifying these parameters changes the way in which the system responds to external perturbations imposed on a given desired posture. In this experiment we would only like to demonstrate how our developmental model can exploit the learned reflex circuits to perform point-to-point trajectories in a way consistent with the TCT [15] (see also [59], [60] for a related model). The experiment is not intended to be a systematic analysis of the performance of the model on this type of tasks, as this will be a matter of future work, but simply to allow the reader to have a broader interpretation of our developmental model.

**Figure 14:**
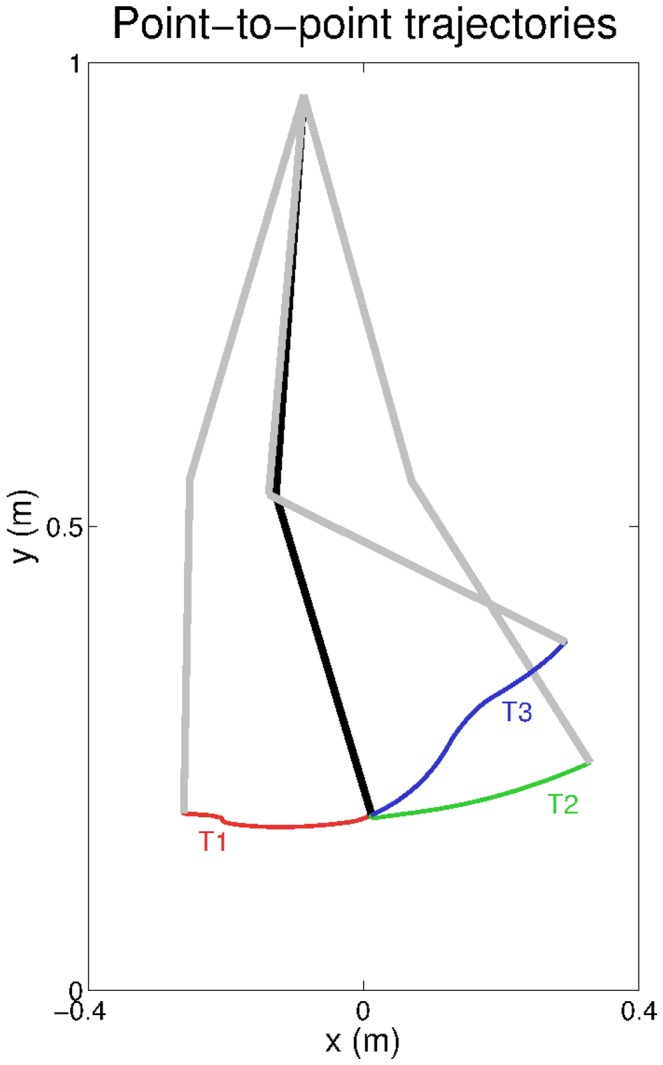
Leg trajectories during point-to-point tasks. The black lines display the initial leg position set for the point-to-point tasks, and the gray lines indicate the leg position achieved at the end of three different trajectories T1, T2, and T3.

The experiment uses the reflexes learnt for the default leg model (see Figure 4). Prior to the experiment, we manually position the leg in three different postures. For each posture, we record the muscle lengths 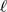 of all the muscles, such that we obtain three sets of muscle lengths 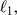 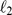 and 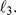 Subsequently, we assign sequentially each of the recorded sets of muscle lengths to the set of desired lengths 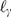 of all the muscles. This assignment produces a change in the active resting length of the muscle, 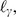 and induces sensory activity in the secondary afferent fibers. This activity, when propagated through the reflex matrix (see eq. 6), leads to a change in the resting position of the leg. The three sets of muscle lengths are applied with the leg in the same initial posture, which is the same as that used in the hopping experiments (see black lines in Figure 14). The 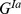 and 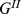 are set manually to minimize the oscillations of the end effector during a given trajectory.

The resulting trajectories are shown in Figure 14 (see also Movie S1.VIII). The figure shows that by using different sets of muscle lengths we can shift the equilibrium posture of the leg, and achieve different end-effector positions. This experiment shows that our model has a natural capability to generate point-to-point movements in a way consistent with TCT (see Introduction).

## Discussion

### Interpretation of the obtained reflex networks

**Figure 15:**
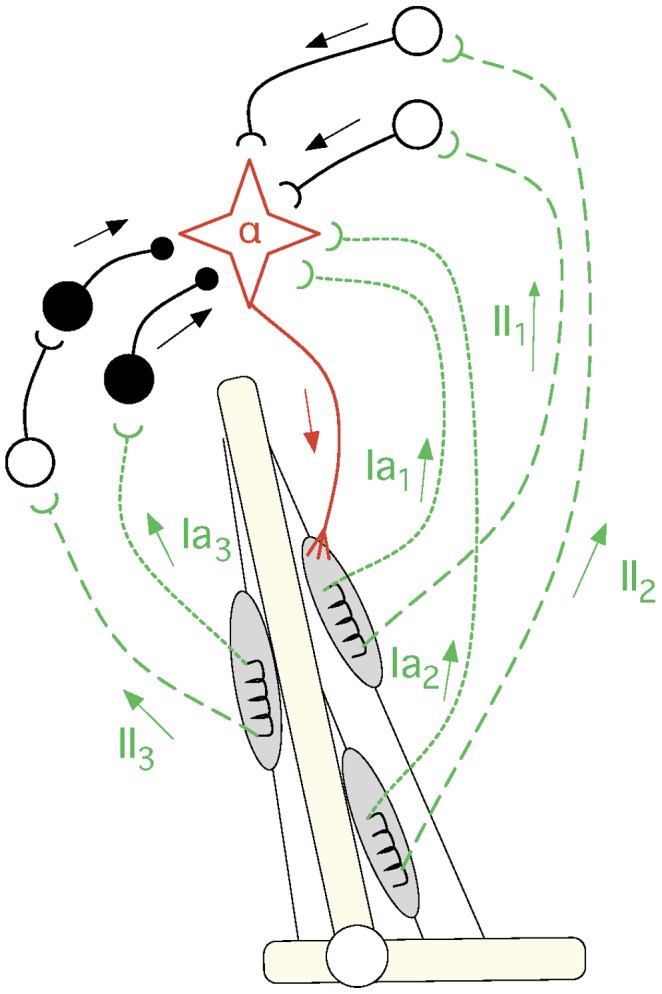
The reflex circuitry involving spindle afferents from primary (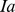) and secondary (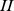) fibres. Filled circles represent inhibitory connections and unfilled circles represent excitatory connections. From a qualitative point of view, the connectivity between both types of afferents (from different muscles) and the *α*-motoneuron of a given muscle is similar (see text). This figure has been adapted from [61].

The mammalian circuitry mediating primary (type-*Ia*) and secondary (type-*II*) spindle afferents, and *α*-motoneurons is shown in Figure 15. Relative to the primary *Ia* afferent fibres [61], [62], the *α*-motoneurons of a given muscle receive direct excitatory connections both from afferents of homonymous (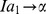) as well as of synergistic muscles (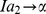). The same muscle receives inhibitory connections from afferents of antagonist muscles through inhibitory interneurons (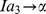). From a functional point of view the connectivity of the secondary afferent is similar to that of the primary afferents, but in the former all the connections seem to be mediated via an additional excitatory interneuron [61].

This circuitry forms the stretch reflex, the purpose of which seems to be to counteract undesired stretches produced by an external load in a given muscle [11] p.439–40. When a load causes a muscle to stretch, it activates both the primary and secondary afferents of that muscle, which in turn recruit the *α*-motoneurons of the homonymous as well as the synergistic muscles [62]–[64]pp.63-6. The activation of both afferents inhibits the antagonist muscles and prevents them from counteracting the movement initiated by the agonist muscles [62]pp.197–200.

The reflexes we obtained with our developmental model are functionally similar to those observed in the mammalian spinal cord (see Figure 4). We obtain excitatory homonymous (e.g. 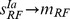 and 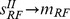 respectively) and synergist connections (e.g. 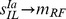 and 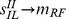), while inhibitory connections are obtained between antagonist muscles (e.g. 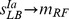 and 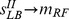). Elsewhere, we have shown that other spinal reflexes can consistently be self-organized using an framework similar to that used here [1], [30], [65] for a mechanical system comprising a pair of agonist-antagonist muscles. We have shown the self-organization of the reverse myotatic reflex, which mediates afferent inputs from the Golgi-tendon organs to *α*-motoneurons, as well as the self-organizion of the withdrawal reflex, which mediates information from cutaneous afferents to *α*-motoneurons (in a way similar to [29]). We have also obtained successfully the reflex circuitry pertaining the type-Ia afferents and motoneurons.

In this context, the work of Petersson and colleagues [29] has been a major source of inspiration for our reflex learning framework. They have observed *in vivo* that by manipulating the sensory information induced by SMA in the context of REM sleep, they could modify the motor responses pertaining the withdrawal reflex. When taken together with our results, this observation raises the question of whether the general organization of spinal reflexes is determined (or at least maintained) by experience-dependent processes induced by SMA, or whether the impact of SMA is restricted to modify only the circuitry pertaining the withdrawal reflex.

When compared with our previous work, this paper presents the following additional contributions. First, it includes the self-organization of reflexes involving type-II afferents, which were absent from previous investigations. Second, it shows that our reflex-learning framework scales to a more complicated (and non-trivial) mechanical system, involving three rigid bodies, two joints and the interactions between six muscles (some of which bi-articular). Third, it shows how the acquired reflexes can be manipulated to orchestrate the activity of the different muscles and thus produce meaningful coordinated behaviour (rhythmic and non-rhythmic). Fourth, it shows that the entire framework can cope naturally with morphological changes.

### The development of spinal reflexes from SMA

One essential component of our developmental model is that of SMA [1]–[3], [36]–[38]. This process consists in the spontaneous activation of *α*-motoneurons, which produce muscle contractions independently of sensory stimulation. SMA has been widely investigated in the chick and the mouse. In this respect chicks often serve as a good model of higher vertebrates because their spinal organization is similar to that of mammals [66]. (Although we should be extremely careful when extrapolating the results from chicks which are a precocial species, to mammals, which can be altricial.)

In chicks and rodents, the patterns of SMA undergo strong changes throughout early development, both pre-natally [66]–[69] and post-natally during REM sleep [39]. In the chick, rhythmic bursts of spontaneous activity in *α*-motoneurons can be recorded by embryonic day 3 (E3), and before motoneuron axons have reached the extrafusal fibres and produce muscle contractions. The frequency of these patterns has been shown to be essential for guiding the axons to reach their appropriate targets [70], [71]. Later, by E7, when the *α*-motoneurons have already established connectivity with muscle tissue, spontaneous muscle contractions seem to occur often alternating the activation of antagonist muscles [67] but without any clear and distinguishable pattern. This is usually termed as type-I motility [72]. Early behavioural analysis has hypothesised an underlying random process [73] (see also [74]), but electromyography (EMG) analysis has shown evidence to the contrary [67]. At this time, muscle afferents start making connections with motoneuron dendrites [75]; such connections seem to be rather coarse and not organized according to any synergist, or antagonist interactions.

With embryonic maturation, clear patterns of SMA seem to progressively emerge. By E9 one can distinguish co-activation of flexor and extensor muscles as well as alternation between antagonist muscles [67]. By E18 stable rhythmic patterns showing the coordination between agonist and antagonist muscles within one limb can be observed [68], [72]. Interlimb coordination seems to be present by E20 [68]. Once the animal is born SMA does not stop; it can still be observed during REM sleep throughout the lifetime of the animal. Like its embryonic counterpart, the patterns of SMA during sleep also seem to undergo a developmental process [39]. Whether all the patterns of SMA observed throughout development involve the same mechanisms seems unlikely. For example, over development brainstem mechanisms seem to be increasingly important for producing twitches [1], which is very unlikely to occur at early embryonic stages (see also [76]–[78] for mechanisms of twitching during sleep).

The spinal cord is not the only place where spontaneous neural activity can be observed; but like spontaneous activity produced in other sites of the brain [79]–[82], SMA has been shown to contribute to a number of developmental processes such as network formation [69], synapse elimination [83]–[85] as well as regulation of synaptic strength [86].

At an initial stage of our project, we hypothesised that SMA could drive the actual formation of reflex circuits, which when fully established, could influence back the process of SMA, and produce more structured patterns of activity such as those observed by E9 in the chick. Although this hypothesis is still theoretically possible, the observation of flexor and extensor synergies as early as E9 in the chick, leaves a very short time frame for experience to drive the formation of reflex circuits (basically from E7–E9). Arguably, the alternation between flexor and extensors observed at E7 is more likely to be produced by some kind of early CPG-like mechanism.

In this context, spontaneous motor activity after birth seems to present more favourable conditions for the kind of experience-dependent reflex learning (or tuning) that we carry out here. First, the muscle twitches occur against a background of muscle atonia. Second, although some muscle synergies can be observed [39], “many of these twitches are dominated by a single muscle” [29]. Third, these twitches have already been shown to modulate the spinal circuitry relative to the withdrawal reflex [29], [87]. Our current hypothesis is that coarse networks are initially established from specific patterns of spontaneous motor activity [69] which are then tuned to the specificities of the particular musculoskeletal system by experience-driven processes (see also [88]).

In this paper, we are not in a position to determine the exact role of SMA in forming, maintaining, or fine-tuning spinal reflex circuits. In this, and other papers, we have only pointed out that the correlation between the sensory and motor signals induced by SMA might contain sufficient information to self-organize a number of spinal reflexes in different sensory modalities. The extent to which such information is actually used in the mammalian system can only be determined empirically.

### Assumptions and implications of the SMA model

Our reflex-learning model takes similar assumptions to those used in Petersson et al [29] to model the tuning of the withdrawal reflex. The only difference is that instead of obtaining stereotypical ipsilateral and contralateral interactions between *α*-motoneurons and tactile sensors, our model extracts stereotypical homonymous, synergist and antagonist interactions between *α*-motoneurons and muscle afferents (in this case spindle afferents). In both models, the order of twitching is not a fundamental factor (see [30] where this order is taken randomly from a uniform distribution), which means that they can handle alternation of antagonists but also alternation of synergist and distal muscles. This can be deduced from the fact that the connections with a given motoneuron 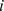 are only updated when 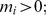 otherwise the they remain unchanged (see eq.5).

Our model, like that of Petersson et al [29], should also be able to deal with some degree of muscle synergies being probabilistically activated simultaneously, independently of whether these synergies consist of co-contraction of antagonists, synergists, flexor or extensors. However, we might obtain some inappropriate reflex connectivity if such synergies are consistently activated, e.g. if two muscles are consistently recruited together. Synergies that are consistently recruited impose a strong bias on the musculoskeletal interactions, which in turn influence the reflex circuitry. The extent to which each synergy can be active for the appropriate reflex circuitry to emerge requires further investigation.

In addition, our SMA patterns are static, i.e. they are influenced only by the activation of the motor elements and by no other factor. This means that reflex circuits do not contribute to the forces present in the system during the passive stage; this is consistent with the observation that spinal reflexes are inhibited during REM sleep [40].

An implication of the SMA process, is that since it provides millions of muscle contractions over the entire lifespan of a mammalian creature it has a great potential to be the driving force underlying the regulation of sensorimotor circuits as we have shown here [1]. In our model this provides one possible explanation for the role of sensory feedback in spinal adaptation [89]. Using our framework, we have shown that modifications in the musculoskeletal system lead the spinal circuits to re-structure (see Figure 10) providing a clear case for motor adaptation. Addressing this hypothesis *in vivo* will be a great challenge that might have a strong impact, not only on the motor control community, but also in the sleep research community. To confirm this hypothesis *in vivo* we will need to carry out experiments in which we analyse how changes in the musculoskeletal system affect known spinal reflex circuits such as the stretch reflex or the withdrawal reflex.

### Assumptions and simplifications of the neural circuitry

The mammalian neural system is plastic and highly redundant, and it is expected that many mechanisms are recruited to achieve complicated behaviours such as hopping, or point-to-point trajectories. In this paper we have simplified to a great extent the overall circuitry of the system. For example, the reflex circuits were modelled using direct connections between sensor receptors and *α*-motoneurons without any mediating interneurons (e.g. Renshaw cells). The supraspinal control was modelled by setting the resting lengths (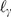) for each muscle, and setting the two reflex gains (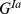 and 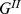). This type of motor control, can be interpreted in two ways. First, it can be interpreted from the point of view of fusimotor control, which assumes that the supraspinal centres are capable of controlling the activity of static and dynamic γ-motoneurons, and thus modulating the sensitivity of type-*II* and type-*Ia* spindle afferents. This type of control is theoretically and experimentally grounded [15], [90], [91] (see also [92]), although the underlying neural circuits have not yet been completely established. And second, it can be interpreted from the point of view of pre-synaptic inhibition [93], [94], in which the reflex synapses modulate the intensity of the reflex responses. Both mechanisms are biologically plausible, and potentially work together. However, whereas fusiform control provides a natural way to account for setting the desired muscle lengths (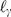) through the activation of γ-motoneurons, pre-synaptic inhibition can only account for the gain modulation.

In addition, the spinal cord contains many circuits other than those addressed in this paper. We neglected the effects of the reverse myotatic and the withdrawal reflexes (which we were already able to develop with our previous framework [30]). This is because, from their connectivity, it was clear that they could only have a marginal role (and probably even counterproductive) during hopping. The interactions between these reflexes could easily be included in our model, but for the sake of simplicity we decided to leave them out.

Behavioural data in cats [95] and humans [96] have shown that under certain conditions reflexes can be reversed to generate positive (instead of negative) feedback reflexes. This has been shown with respect to circuits comprising spindle 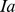 afferents [7], [97] as well Golgi-tendon 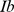 afferents [95], [96], [98]. This reflex reversal is not achieved by reverting the nature (excitatory or inhibitory) of the established reflex circuits, but rather through oligosynatpic networks which coexist in parallel to these circuits [97]. It has been hypothesised that switching between the positive and the negative feedback circuits is carried out by modulating the reflex gains of the respective networks to favour the expression of ones or the others [97].

From a functional point of view, force (as well as length) positive feedback loops can be helpful in providing extra force to the system where other mechanisms might be unable to do so. For example, in the context of our paper, we are not imposing any limitations on the forces provided by the stretch reflex circuits, since we do not impose any upper limit on the reflex gains. In the mammalian system, this is a rather implausible assumption, and often the system might need to recruit force from additional sources other than those that can be provided by negative feedback loops (as the stretch reflex) alone. Positive feedback circuits are a good candidate mechanism to provide such forces.

In our developmental framework, it would be possible (in principle) to obtain both types of feedback loops (negative and positive) in a consistent way, if in addition to the anti-Oja rule we allow for other networks to be defined by the standard version of the Oja-rule [41]. Critically, the balancing of the additional reflex networks could then be achieved through a gain modulation process similar to that described in this paper. At this stage of our research, we do not see a direct benefit of adding positive force reflex networks to our model, since we could achieve the desired behaviours without them. However, for a more realistic modelling of the spinal circuitry, such circuits would have to be taken into account.

### Reflexes as possible mechanisms to coordinate behaviour

In mammals, there are a number of circuits that contribute to the observed patterns of coordinated behaviour. These range from reflex and central pattern generator (CPG) circuits in the spinal cord [99], [100], to more central circuits located in the brainstem [6], cerebellum and cortical areas [101], [102]. From a locomotor perspective, the roles of reflexes and CPGs have been historically disputed. On the one hand, the observation that deafferented mammals – including humans – can produce locomotor patterns seemed to undermine the contribution of reflexes during locomotion [103], [104]. In these animals, the rhythmic motion was governed mainly by CPG networks located in the brainstem and spinal cord [6]. On the other hand, the observation that reflexes are modulated during the locomotor cycle, suggests that they are an intrinsic part of coordinated locomotor behaviour [55], [56]. Currently, it is commonly agreed that reflexes are essential to achieve the full locomotor capabilities observed in mammals, which take into account adaptability to environmental perturbations as well as load bearing [8]–[10].

In our developmental model, we have deliberately left out any circuits pertaining CPGs. Although we are using a rythmic behaviour (i.e. hopping) as the main case study for coordinated behaviour, our framework is intended to have a broader interpretation, which is not restricted to periodic locomotor patterns. In general, our hypothesis is that the coordinated motor responses provided by reflexes go beyond a mechanism that deals only with individual perturbations imposed by the environment. Our hypothesis is that reflexes provide training signals (either directly, or through the induced sensory stimulation) that can be incorporated in the learning of new behaviours or in the modification of existing ones. This is not intended to dispute, nor undermine, the role of CPGs in producing locomotor behaviours; only to show that, in principle, reflexes also have an inherent capacity to coordinate behaviour. Such capacity can potentially be used to modify the activity of CPG circuits involved in locomotion, but also circuits pertaining other behaviours which are not necessarily connected with locomotion (e.g. point-to-point trajectories). For this to be possible reflexes have to: 1) already provide some basic form of coordinated muscle activity, and 2) be modifiable to achieve some desired criterion. Although this is not a completely new idea (e.g. [17]), it is one of the aspects we would like to highlight in our paper.

In addition, we would like to note that to achieve rhythmic behaviours that require repeated shifts in the body posture, like walking or running, we would actually need to extend the model to incorporate a CPG-like mechanism. Such a mechanism would not only be responsible for setting the different leg postures, 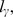 during the locomotor cycle, but it could also modulate the reflex circuits in time as we have shown in the context of behavioural transitions (see also [105]). This is currently one of the challenges we are addressing with our model.

### Implications of the mechanical muscle model

Our muscle model consists of a spring and damper systems in parallel with a contractile unit. In this paper we do not model the elasticity provided by tendons. Whereas the effect of tendon elasticity would have a negligible effect during the passive stage (i.e. during reflex learning), it could potentially influence the final hopping behaviour, since the latter entails much larger forces than the former. Tendon-elasticity has been shown to contribute to locomotor behaviours in several ways [106], [107]. First, it provides passive energy storage and recovery, which allows for higher energy efficiency; second, it allows to recoil the muscle-tendon complex faster than the muscle alone. In this paper, we are mainly investigating the effectiveness of the learned reflex circuits in coordinating the overall muscle activity during the hopping behaviour, without any further considerations on the actual maximum muscle forces. In principle, incorporating muscle elasticity in our model should allow to reduce the overall muscle activity during hopping, and thus increase the energy efficiency (a measure that falls out of the scope of this paper). However, we would not expect a significant difference on the capability of the reflexes to orchestrate the activity of the different muscles, provided that the gains 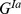 and 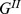 can be modified to accommodate the new dynamics of the system. This hypothesis will need to be addressed experimentally in future work.

### Implications of the gain modulation mechanisms

In our system, the reflex gains have been identified manually. This process of identification resembles that of tuning a general PD-Controller, i.e. a controller of the form 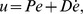 where 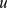 is the output of the controller, 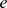 is the error between the current value of the variable being analysed and a given desired reference value, and 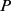 and 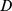 are constant parameters. The main difference here is that the variable that is being measured is not the same as the variable that is being used by the controller to produce motor activity. In our model, we use the hopping height to measure the quality of the coordinated behaviour, but the controller uses local information to produce the actual muscle activations (i.e. muscle deformation and velocity). This is consistent with the idea that manipulating reflex circuits leads to different equilibrium points (or trajectories) without necessarily encoding them explicitly [15].

One aspect of the framework that needs to be verified is the generality of modulating reflex networks for the entire body, in contrast to single joints. We have recently equipped our leg with an upper torso as well as with the muscles required to balance it, and we have tested its capability to hop. Our preliminary results show that hopping and the balancing of the torso can be achieved simultaneously with a single pair of gains (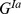 and 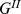), but we have not yet managed to achieve a stable hopping pattern with this system (see also [108] for common muscle synergies during walking and balancing). We suspect that the reflex networks that govern the stabilization of the upper torso should be modulated independently from those that coordinate the leg muscles during the hopping, but so far we have not yet managed to frame such a separation in a developmental way.

### Muscle synergies, modularity, and reduction of dimensionality

In our framework the muscle synergies are given by the reflex networks, which for a given pattern of sensory stimulation specify the activation profiles of all the different muscles. We believe that this is an important contribution since it realises the concept of muscle synergy at the neural level, and it shows how such synergies can emerge out of musculoskeletal interactions. In doing so we provide a specific and testable model which can be validated or falsified.

Mathematically, our synergies can be compared with those in [108], which consist of a linear combination of muscle activations; the only difference being that the temporal pattern of the synergy is given by the sensory input rather than being fixed. It must be noted that this pattern of sensory input cannot be just any pattern. Artificially induced afferent activity does not necessarily lead to motor coordination from reflex circuits. This has been shown, for example by Klein et al [109] in rats; when stimulating the fifth lumbar dorsal roots they observed periodic patterns of motor activity which were closer to CPG-like mechanisms rather than reflex responses. This observation contrasts with more localized and natural afferent stimulation. For example, the stimulation of spindle afferents of a given muscle produces a well known reflex-like response that resembles the stretch reflex (this is usually termed the H-reflex). This type of stimulation simulates activity of the spindles when a stretch is imposed on the muscle, and thus produces a more natural reflex-like response in the animal. In our model, when the leg touches the ground it also produces a consistent set of afferent inputs in which the extensor spindles are active but not flexor spindles. Such a natural and consistent pattern of sensory stimulation is essential for the induced reflex activity to be able to produce a coordinated motor response.

The issue of sensory stimulation, brings us to one of the challenges faced by our model; without sensory inputs there is no muscle coordination (or muscle synergies). This contrast heavily with natural observations in deafferented mammals, which can still carry out coordinated behaviour in the absence of sensory stimulation [21].

From a developmental prespective, this type of observation needs to be carefully contextualised. Let us take the example of the Vestibulo-Ocular Reflex (VOR), which produces contractions in the muscles of the eyes to compensate for rotations in the head (as sensed by the vestibular system). As the VOR can be triggered in the dark [110] and can be observed in subjects who developed blindness [111], one might be tempted to argue that vision is not a relevant stimulus for this reflex. In fact, the circuitry of the reflex confirms this hypothesis, since it contains only connections mediating vestibular and oculomotor elements. However, studies have shown that subjects who are congenitally blind do not develop the VOR, which brings a new perspective onto the original hypothesis [111].

This example shows that observations in adults are often insufficient to infer whether a certain neural element (in our case the sensory inputs) is required or not for a particular behaviour. As in the VOR, spinal and supraspinal systems could be entrained by the continued exposure to the motor patterns produced in the context of a given behaviour (say, walking), and could then be capable of reproducing it after in the absence of sensory stimulation. This hypothesis makes a case for the importance of development to understanding motor control (see for example, [16], [28], [112], [113]); but, unfortunately we are not yet at a stage where we can address it with our model.

In addition, our model allows us to make a case for dimensionality reduction. The dimensionality of our system can be measured in a number of ways. The system can be thought to have 6 dimensions (the number of muscles to be actuated), 18 dimensions (6 motor outputs + 12 sensor inputs), or 72 dimensions (i.e. the number of reflex arcs, 6 motor outputs 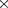 12 sensor inputs). In spite of this, we control only two variables, i.e. 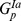 and 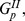 which manipulate the reflex circuits at the network level, rather than control individual elements of the system (like motor outputs, or single reflex arcs). This is only possible because the self-organized reflex circuits already encode meaningful musculoskeletal interactions, and equip the system with a mechanism that coordinates the muscle activity at a local level. Although we do not know exactly how the modulation mechanisms of the central nervous systems work, our model provides a candidate mechanism to realize them.

### Implications of the developmental model in artificial systems

We believe that our developmental model can also contribute to the domain of artificial systems and robotics. The recent increase in the complexity of artificial systems (i.e. systems endowed with large number of sensor and motor elements [114]–[118]) is propelling research to identify novel methods that can automatize the process of gathering meaningful information about the body morphology. In this context, SMA can be taken as an analogue to the impulses used in control theory for the purposes of system identification, where they are typically applied to a system in order to characterise (and model) its input-output relations [119] (see also [29]). In addition, forms of SMA have also been used in real and simulated systems to structure sensorimotor information [120], [121]. Like in our reflex circuits, the sensorimotor signals are coupled according to some form of correlation measure; often some measure taken from information theory [120]. However, the correlated signals are typically exploited for some form of perception [121], or prediction [122], unlike our reflex circuits which are exploited to coordinate behaviour directly.

In this respect, we believe that to make our developmental model fully applicable in an artificial system, we would need to automate the process of learning the reflex gains, which is currently carried out manually. This is out of the scope of the this paper, but it is a feature we are planning to address in future work. Such a learning mechanism could have the benefit of removing the human out of the control loop, and allow the artificial system to automatically acquire information about its body (through the learning of reflexes) and exploit it to produce coordinated behaviour.

Our developmental model can also contribute to the field of locomotion of artificial systems. In this context, one of the most widely used models is the Spring Loaded Inverted Pendulum (SLIP). This model consists of a point-mass system connected to the ground via a spring. In its original formulation the model assumes the conservation of energy [123] (i.e. it assumes no energy losses due to contacts with the ground) but such an assumption has been relaxed in subsequent extensions [124]. The SLIP model has been used (and extended) to analyse energy efficiency and behavioural stability during running [123], walking [125], hopping [126] as well as during behavioural transitions [127].

In this context, the model of Shafarbi and colleagues [126] provides a good comparison with our model as it addresses directly hopping. Their model, which they called XTSLIP, extends the SLIP model by including an upper torso and a leg (both with mass). The leg consists of a spring and a damper system, which produce forces when perturbed during the contact with the ground. The control of the hopping height is done by changing the resting length of the spring during the stance phase. Some of the premisses of our model resonate with those in the XTSLIP. First, in both models hopping emerges out of the perturbations induced by the ground to the equilibrium position of the system. Second, in our model the muscle activations produced in the context of the 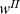 network (which deals with information relative to muscle deformation) can be seen as an analogue of the mechanical spring in the XTSLIP, the stiffness of which can be controlled by the gain 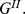 The main difference is that the equilibrium position in our system is maintained via a set of negative feedback loops, and in the XTSLIP it is abstracted in a purely mechanical system.

In the XTSLIP, energy losses are dealt with by controlling the resting length of the spring during the stance phase. This modifies the equilibrium point of the system and allows energy to be pumped into it. In our model, the set of desired muscle lengths (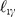) can be interpreted as the resting length of the spring in the XTSLIP. We have shown that by modifying these desired lengths we can change the equilibrium point of our system and obtain different leg postures (see 14). However, during hopping the set of desired muscle lengths is kept constant. In contrast, our strategy to pump energy during hopping consists in modulating the network 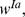 which deals with information relative to muscle velocity (i.e. the rate of change in the muscle lengths). This strategy can be compared with that of McGeer in his extension to the SLIP model [124].

## Supporting Information

Figure S1 **Hinton diagrams of the reflex circuits obtained with the default leg model using a twitching amplitude of m = 10.** a) Circuits obtained for the *Ia*-type afferents, and b) those obtained for the *II*-type afferents. Unfilled circles represent excitatory connections, and filled circles represent inhibitory connections. Note that although some of the connections have changed, the general connectivity between homonymous (in the diagonal), antagonist, and synergist muscles are kept.
(TIF)

Figure S2
**The hip trajectory and the mean and standard deviation of the kinematic and dynamic variables obtained using the reflex matrices resulting from a twitching amplitude of m = 10.** a) Kinematic and dynamic variables obtained using the reflex matrices resulting from a twitching amplitude of m = 10. b) The hip trajectory recorded for the new reflex matrices. Although some oscilations can be observed during the flight phase, we can obtain a very stable hopping pattern, 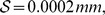 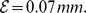

(TIF)

Figure S3
**The hip trajectory and the mean and standard deviation of the kinematic and dynamic variables obtained for the different ground models.** Kinematic and dynamic variables obtained for the system with a) ground model 1, 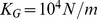 and 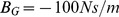 (hopping stability 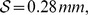 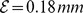), b) ground model 2, 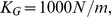 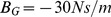 (hoppings stability, 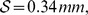 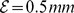), c) ground model 3, 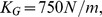 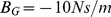 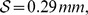 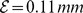). The hip trajectory recorded for the system with d) ground model 1, e) ground model 2, and f) ground model 3.(TIF)

Figure S4
**The hip trajectory and the mean and standard deviation of the kinematic and dynamic variables obtained for system with modified mass.** a) Kinematic and dynamic variables obtained for the system with 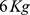 (hopping stability 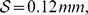 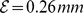). b) The hip trajectory recorded for the system with 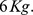

(TIF)

Figure S5
**The hip trajectory and the mean and standard deviation of the kinematic and dynamic variables obtained for the a biological muscle model.** a) Kinematic and dynamic variables obtained for the muscle model 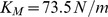 and 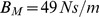 ([35]), and b) the hip trajectory recorded for the the biological muscle model. As can be observed we can also achieve a very stable hopping pattern with the biological muscle model parameters (Although some oscilations can be observed during the flight phase, we can obtain a very stable hopping pattern, 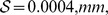 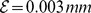).(TIF)

Movie S1
**Video of the different experiments carried out in the paper.** I) behaviour observed during SMA, II) behaviour of the leg when 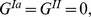 III) unstable hopping, IV) stable hopping, V) stable hopping with a changing ground, VI) stable hopping after changing the attachment points of the 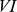 muscle, VII) behavioural transition, VIII) point-to-point trajectories.(MP4)
