# Supplementary figures and images for: From Spontaneous Motor Activity to Coordinated Behaviour: A Developmental Model
Source: PLoS Comput Biol. 2014 Jul 24;10(7):e1003653. doi: 10.1371/journal.pcbi.1003653 (PMC4109855; doi:10.1371/journal.pcbi.1003653)

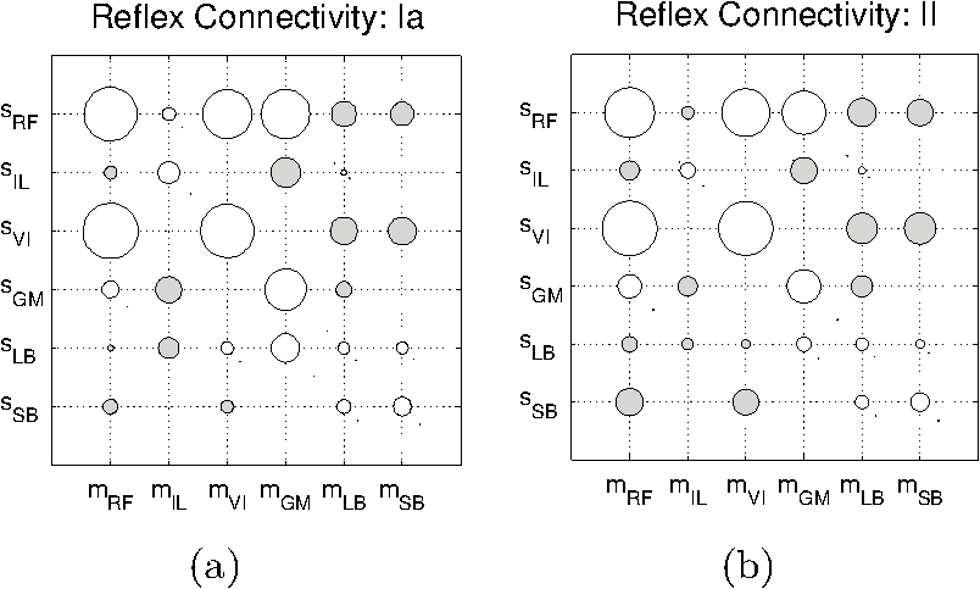

Supplement: Figure S1 — Hinton diagrams of the reflex circuits obtained with the default leg model using a twitching amplitude of m = 10. a) Circuits obtained for the Ia-type afferents, and b) those obtained for the II-type afferents. Unfilled circles represent excitatory connections, and filled circles represent inhibitory connections. Note that although some of the connections have changed, the general connectivity between homonymous (in the diagonal), antagonist, and synergist muscles are kept. (TIF) [file pcbi.1003653.s001.tif]

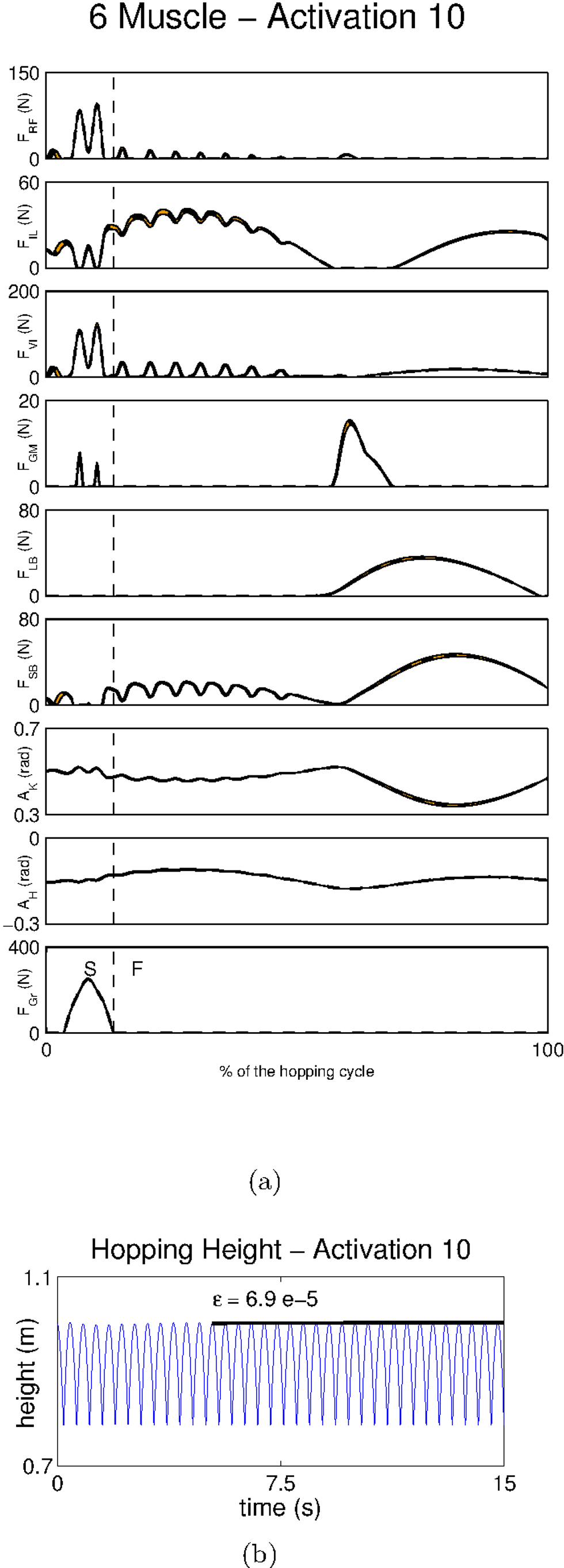

Supplement: Figure S2 — The hip trajectory and the mean and standard deviation of the kinematic and dynamic variables obtained using the reflex matrices resulting from a twitching amplitude of m = 10. a) Kinematic and dynamic variables obtained using the reflex matrices resulting from a twitching amplitude of m = 10. b) The hip trajectory recorded for the new reflex matrices. Although some oscilations can be observed during the flight phase, we can obtain a very stable hopping pattern, (TIF) [file pcbi.1003653.s002.tif]

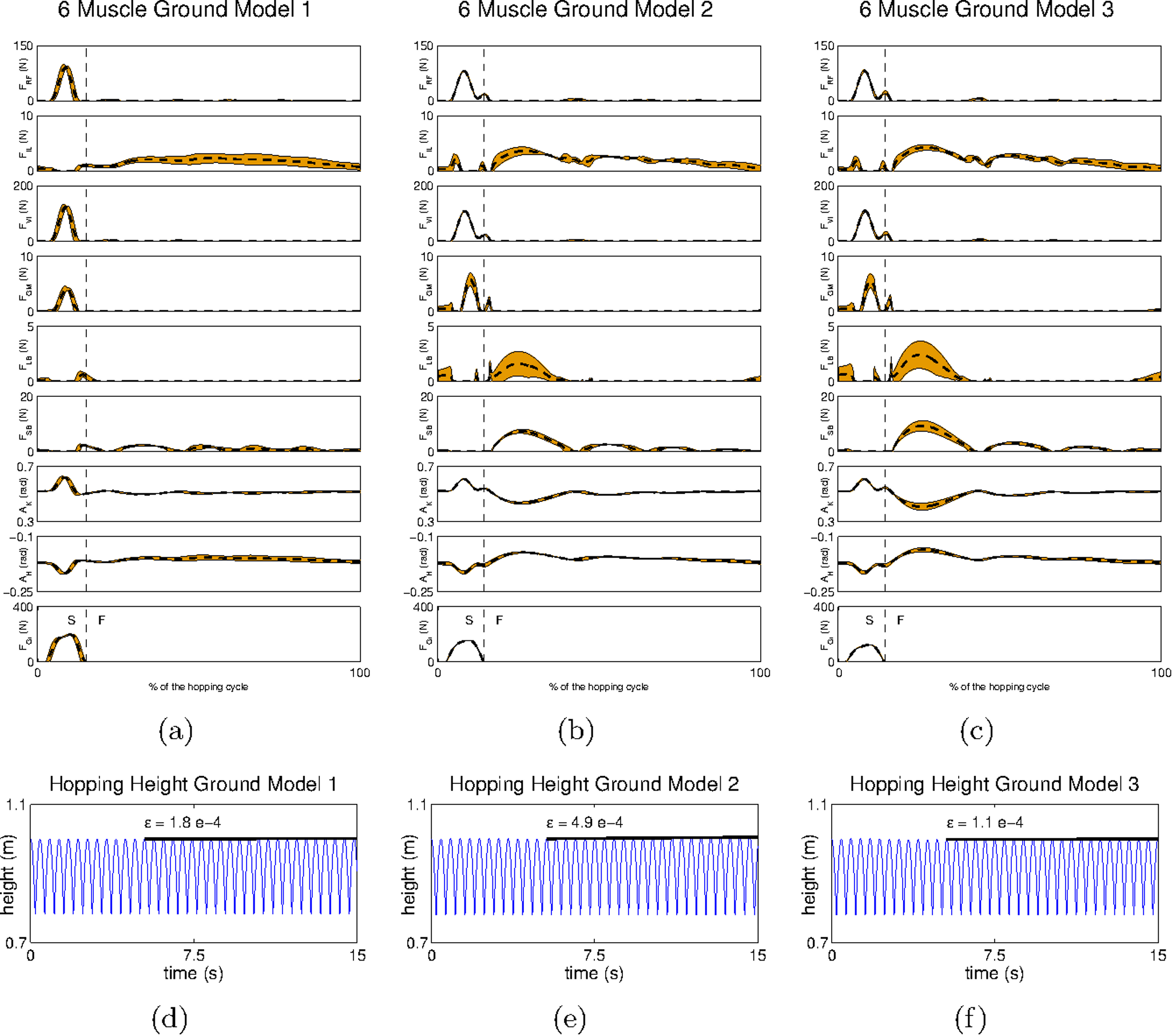

Supplement: Figure S3 — The hip trajectory and the mean and standard deviation of the kinematic and dynamic variables obtained for the different ground models. Kinematic and dynamic variables obtained for the system with a) ground model 1, and (hopping stability ), b) ground model 2, (hoppings stability, ), c) ground model 3, ). The hip trajectory recorded for the system with d) ground model 1, e) ground model 2, and f) ground model 3. (TIF) [file pcbi.1003653.s003.tif]

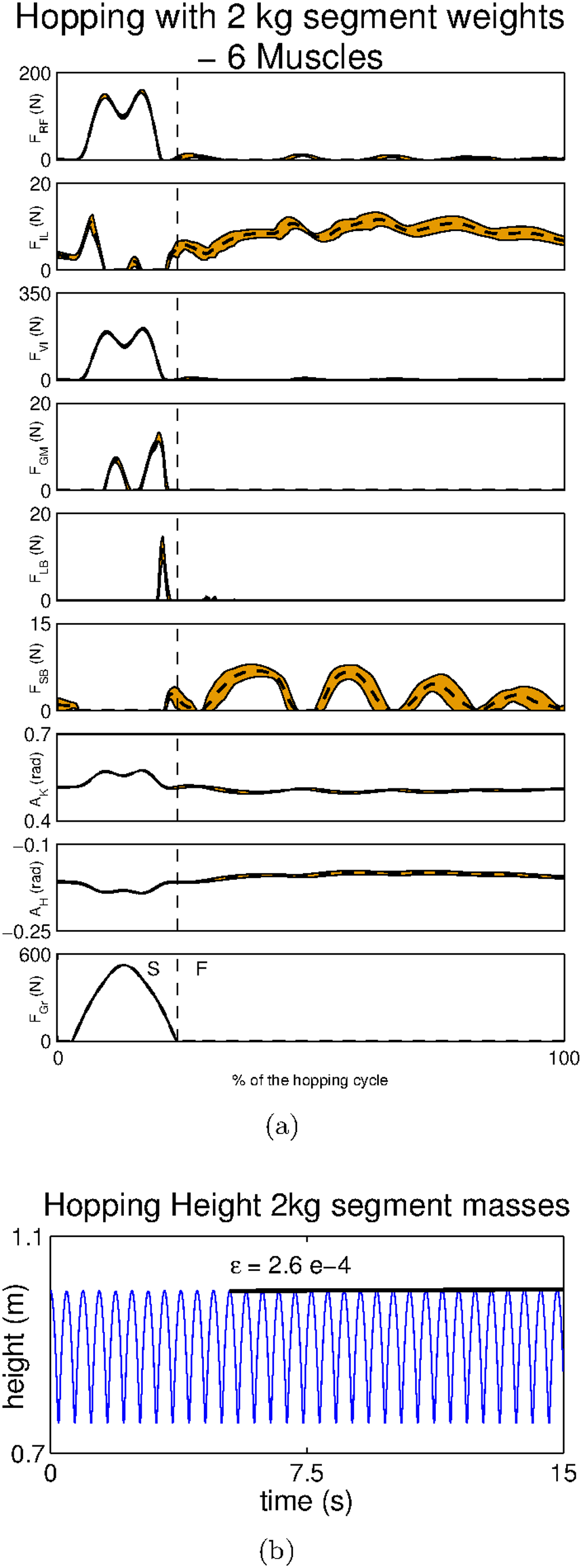

Supplement: Figure S4 — The hip trajectory and the mean and standard deviation of the kinematic and dynamic variables obtained for system with modified mass. a) Kinematic and dynamic variables obtained for the system with (hopping stability ). b) The hip trajectory recorded for the system with (TIF) [file pcbi.1003653.s004.tif]

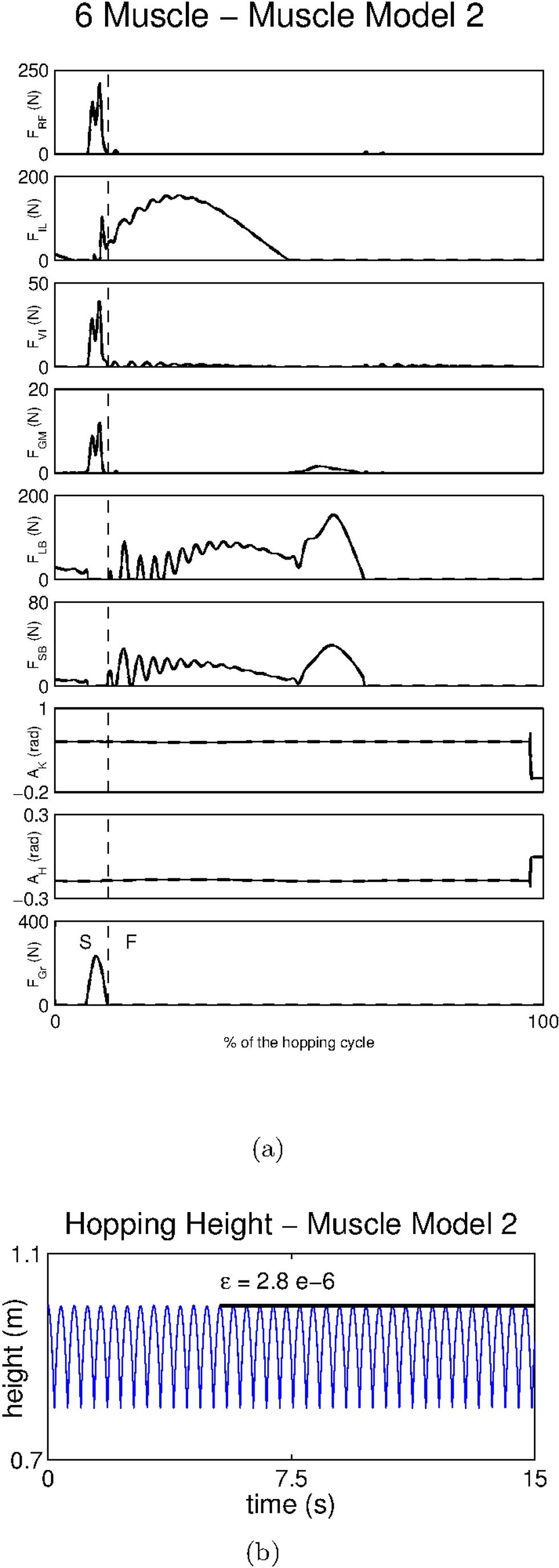

Supplement: Figure S5 — The hip trajectory and the mean and standard deviation of the kinematic and dynamic variables obtained for the a biological muscle model. a) Kinematic and dynamic variables obtained for the muscle model and ([35]), and b) the hip trajectory recorded for the the biological muscle model. As can be observed we can also achieve a very stable hopping pattern with the biological muscle model parameters (Although some oscilations can be observed during the flight phase, we can obtain a very stable hopping pattern, ). (TIF) [file pcbi.1003653.s005.tif]
